# Functional and structural characterization of *Streptococcus pneumoniae* pyruvate kinase involved in fosfomycin resistance

**DOI:** 10.1016/j.jbc.2023.104892

**Published:** 2023-06-05

**Authors:** Atsushi Taguchi, Ryosuke Nakashima, Kunihiko Nishino

**Affiliations:** 1 SANKEN (The Institute of Scientific and Industrial Research), Osaka University, Ibaraki, Osaka, Japan; 2 Graduate School of Pharmaceutical Sciences, Osaka University, Suita, Osaka, Japan; 3 Center for Infectious Disease Education and Research, Osaka University, Suita, Osaka, Japan

**Keywords:** pyruvate kinase, antibiotic resistance, enzyme kinetics, allosteric regulation, *Streptococcus*, enzyme structure, fosfomycin

## Abstract

Glycolysis is the primary metabolic pathway in the strictly fermentative *Streptococcus pneumoniae*, which is a major human pathogen associated with antibiotic resistance. Pyruvate kinase (PYK) is the last enzyme in this pathway that catalyzes the production of pyruvate from phosphoenolpyruvate (PEP) and plays a crucial role in controlling carbon flux; however, while *S. pneumoniae* PYK (*Sp*PYK) is indispensable for growth, surprisingly little is known about its functional properties. Here, we report that compromising mutations in *Sp*PYK confers resistance to the antibiotic fosfomycin, which inhibits the peptidoglycan synthesis enzyme MurA, implying a direct link between PYK and cell wall biogenesis. The crystal structures of *Sp*PYK in the apo and ligand-bound states reveal key interactions that contribute to its conformational change as well as residues responsible for the recognition of PEP and the allosteric activator fructose 1,6-bisphosphate (FBP). Strikingly, FBP binding was observed at a location distinct from previously reported PYK effector binding sites. Furthermore, we show that *Sp*PYK could be engineered to become more responsive to glucose 6-phosphate instead of FBP by sequence and structure-guided mutagenesis of the effector binding site. Together, our work sheds light on the regulatory mechanism of *Sp*PYK and lays the groundwork for antibiotic development that targets this essential enzyme.

*Streptococcus pneumoniae* is a Gram-positive opportunistic pathogen often found in respiratory tract infections, and it is known to cause severe diseases such as pneumonia, meningitis, and sepsis (1). In particular, the recent increase in the number of deaths associated with multidrug-resistant *S. pneumoniae* makes it one of the top-priority pathogens for antimicrobial research (2). A better understanding of pneumococcal physiology would facilitate the development of effective treatments against drug-resistant strains. In contrast to cellular processes that are targeted by commonly used antibiotics, those related to central metabolism have received relatively little attention. *S. pneumoniae* metabolism is defined by the lack of the tricarboxylic acid cycle and the respiratory electron transport chain, making it a strictly fermentative bacterium that relies on carbohydrate acquisition for energy generation (3). *S. pneumoniae* possesses a diverse set of glycosidases and transporters for sugar import, which enables its growth within various host environments. The imported products are converted to glycolytic intermediates that are processed through glycolysis to generate pyruvate, which is further metabolized either by homolactic fermentation or by mixed acid fermentation (4, 5). Carbohydrate metabolism is inherently linked to other biological pathways such as amino acid biosynthesis and capsule biosynthesis, and coordination of these pathways is crucial for pneumococcal survival and fitness (6, 7).

A key enzyme that plays a central role in metabolic flux is pyruvate kinase (PYK), which catalyzes the last step in glycolysis by converting phosphoenolpyruvate (PEP) and ADP to pyruvate and ATP. Unlike a subset of bacterial species (*e.g. Escherichia coli*, *Pseudomonas aeruginosa*) that possesses two PYK isoforms, *S. pneumoniae* encodes a single *pyk* gene that has been annotated as indispensable for survival in genome-wide essential gene analyses (8, 9, 10, 11). From previous studies, it is well-established that PYKs adopt a homotetrameric structure and are typically regulated allosterically by sugar phosphates, although the regulation mechanism appears to vary significantly among different species and has been the topic of ongoing research (12). For example, *E. coli* PykF is strongly activated by fructose 1,6-bisphosphate (FBP) while the *P. aeruginosa* isozyme is stimulated by glucose 6-phosphate (G6P) (13, 14). PYK activity has been shown to affect DNA replication and cell division processes in the model Gram-positive bacterium *Bacillus subtilis*, highlighting the importance of regulating its activity for cell proliferation (15, 16). Surprisingly, no detailed characterization of *S. pneumoniae* PYK (*Sp*PYK) has been reported to date despite the outstanding significance of glycolysis in pneumococcal growth and its anticipated role in mediating crucial cellular processes.

While investigating the drug resistance mechanisms of *S. pneumoniae*, we serendipitously discovered *pyk* mutants that confer resistance to the phosphonic acid antibiotic fosfomycin, which has been used clinically for treating urinary tract infections (17, 18). Fosfomycin targets MurA, which is the first enzyme in the peptidoglycan synthesis pathway that catalyzes the transfer of the enolpyruvate moiety from phosphoenolpyruvate (PEP) to UDP-N-acetylglucosamine (Fig. 1). This product is then converted to UDP-N-acetylmuramic acid, which is used to synthesize the peptidoglycan precursor Lipid II. Fosfomycin acts as a PEP analog and inhibits MurA by covalently binding to the active site cysteine (19). *S. pneumoniae* possesses two MurA homologs, MurA1 and MurA2, which are both inhibited by fosfomycin (20). Its efficacy against pneumococcal infections has been demonstrated *in vivo*, especially in combination with other antibiotics that target peptidoglycan synthesis (21). Two major mechanisms of fosfomycin resistance have been studied extensively in other organisms (17, 18). In *E. coli*, fosfomycin uptake is mediated by the glycerol-3-phosphate transporter GlpT and the glucose-6-phosphate transporter UhpT, and mutations in these transporters confer fosfomycin resistance. Some bacterial species are known to possess metalloenzymes that inactivate fosfomycin by catalyzing its epoxide ring-opening. However, *S. pneumoniae* does not encode any of these transporters or fosfomycin-inactivating enzymes, and how it acquires fosfomycin resistance remained unknown (22).

**Figure 1:**
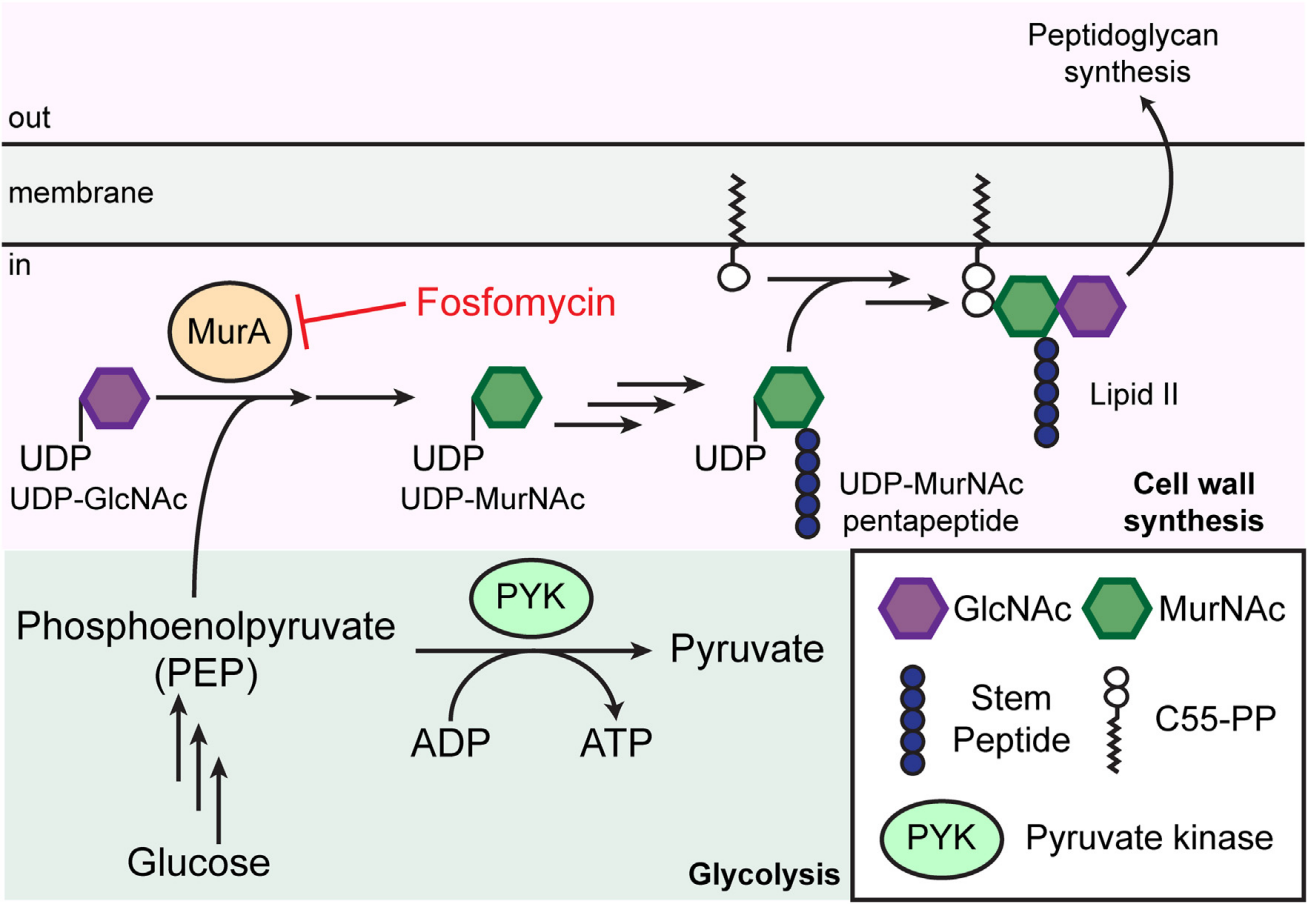
**Schematic of the glycolysis and peptidoglycan synthesis pathways.** Phosphoenolpyruvate (PEP) is an intermediate in the glycolysis pathway that is converted to pyruvate by pyruvate kinase (PYK). PEP is also used by MurA, which catalyzes the first committed step in peptidoglycan precursor (Lipid II) synthesis. Fosfomycin exerts its antimicrobial activity by covalently binding to MurA and inhibiting cell wall synthesis. C55-PP: undecaprenyl pyrophosphate.

In this study, we present evidence that *Sp*PYK mediates metabolic flux into the peptidoglycan synthesis pathway by showing that lower *Sp*PYK activity results in elevated fosfomycin resistance. We also show crystal structures of *Sp*PYK containing the physiological substrate PEP and allosteric activator FBP in different combinations, which provide insight into the allosteric activation mechanism. Notably, we discovered that FBP in *Sp*PYK binds to a location distinct from the FBP binding site in human M2PYK, which has garnered significant interest in recent years in the context of oncogenesis (23, 24). Furthermore, we demonstrate that G6P-activated *Sp*PYK could be engineered by introducing sequence and structure-guided mutations in the effector binding site. This work advances our understanding on the functional and regulatory characteristics of *Sp*PYK and provides a basis for developing PYK inhibitors that could be used to treat pneumococcal infections.

## Results

### Fosfomycin-resistant *S. pneumoniae* isolates contain compromising *pyk* mutations

**Figure 2:**
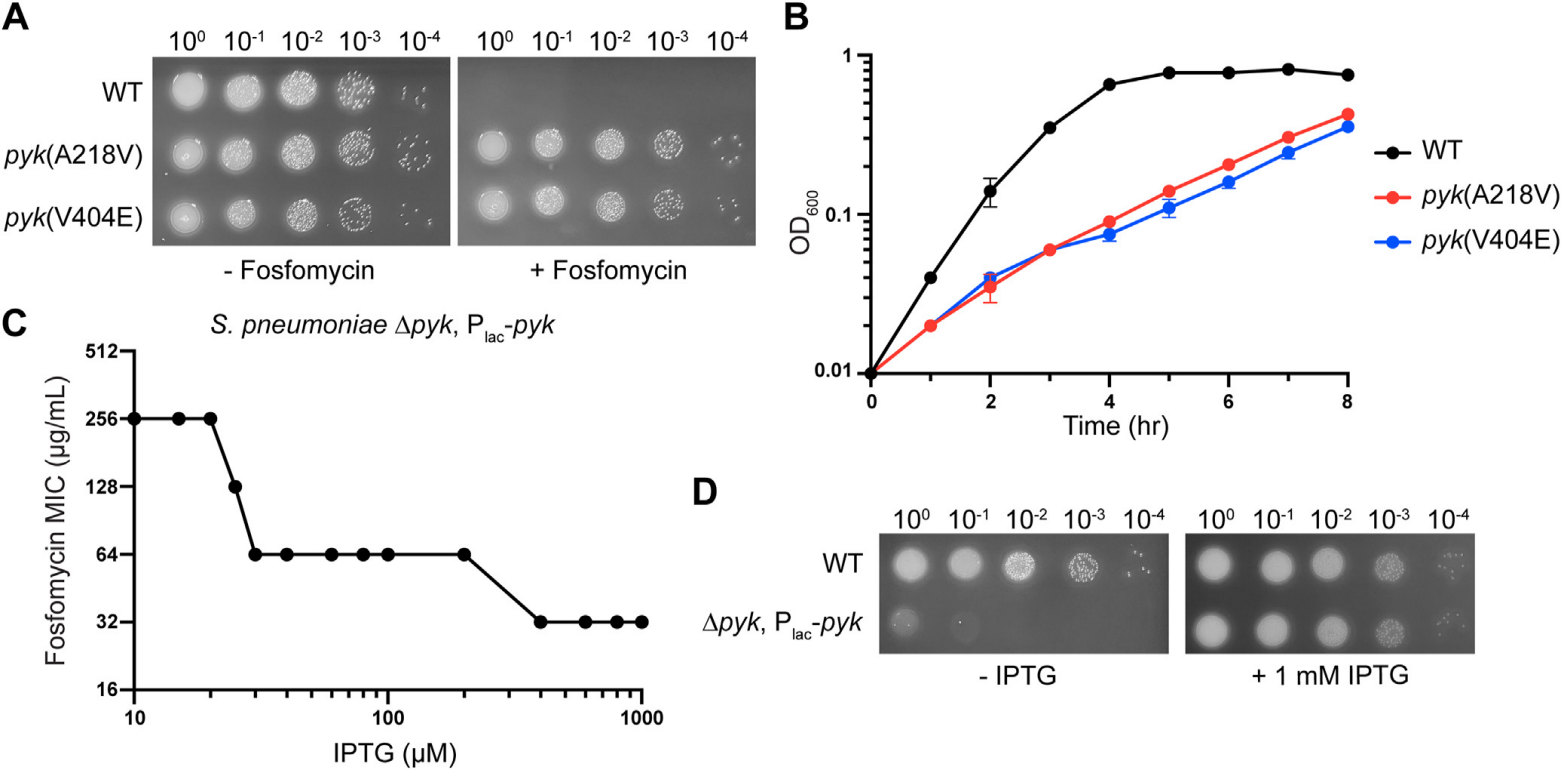
**Lower pyruvate kinase activity results in slower growth and higher fosfomycin resistance in *Streptococcus pneumoniae*.***A*, *S. pneumoniae* D39 wild-type (WT) and isolates with the indicated mutation in PYK were spotted on an agar plate with fosfomycin (64 μg/ml) to confirm the fosfomycin resistant phenotype. These isolates also contain an additional mutation in DltD (See Table S1). *B*, representative growth curves of D39 WT and fosfomycin resistant isolates. Error bars represent mean ± SD from duplicates. *C* and *D*, Fosfomycin MIC measurement and spot dilution assay of *S. pneumoniae* R6 Δ*pyk* strain containing an ectopic *pyk* under an IPTG-inducible promoter (P_lac_). This strain also expresses LacI which prevents leaky expression of P_lac_ (27).

We initially sought to understand how *S. pneumoniae* becomes resistant to fosfomycin by identifying genetic mutations involved in this phenotype. To obtain resistant mutants, we plated the serotype 2 encapsulated strain D39 and its unencapsulated derivative laboratory strain R6 on blood agar plates containing lethal concentrations (D39: 128 μg/ml; R6: 256 μg/ml) of fosfomycin and obtained small colonies of resistant mutants following a 40 h incubation. We selected two isolates from each strain for characterization by whole genome sequencing, which all contained point mutations in the pyruvate kinase (PYK) gene (D39: A218V or V404E; R6: T407A or D393Y) (Table S1). In addition, D39 isolates contained a frame-shift mutation or a point mutation in *dltD*, which is a member of the *dlt* operon that encodes proteins required for D-alanylation of teichoic acids (Table S1) (25, 26). Acquisition of stable resistance was confirmed by a spot-titer assay on solid media containing fosfomycin, and minimum inhibitory concentration (MIC) measurements in liquid media revealed an 8 to 16-fold increase in fosfomycin MIC values compared to those obtained for progenitor strains (Figs. 2*A*, S1 and Table S1). We measured the growth rate of fosfomycin-resistant isolates and found that these isolates grew slower than their wild-type counterparts, which indicated that PYK mutations in these isolates have compromising effects on PYK activity (Figs. 2*B* and S2).

We hypothesized that if the reduction in PYK activity contributes to fosfomycin resistance, then cells expressing lower levels of wild-type PYK would be less susceptible to this antibiotic. To test this hypothesis, we constructed a Δ*pyk* strain containing an ectopic copy of the *pyk* gene under an isopropyl β-D-1 thiogalactopyranoside (IPTG)-inducible promoter that has been shown to display a dynamic induction range (27). We proceeded to measure the fosfomycin sensitivity of this strain under different IPTG concentrations and found that lower PYK expression corresponds to higher fosfomycin MIC (Fig. 2*C*). This strain was unable to grow on solid media lacking IPTG, confirming that PYK is essential for *S. pneumoniae* growth in a standard laboratory environment (Fig. 2*D*). Taken together, these results show that PYK activity is inherently linked to fosfomycin resistance in *S. pneumoniae*.

### *Sp*PYK is a potassium-dependent allosteric enzyme that is activated by FBP

**Figure 3:**
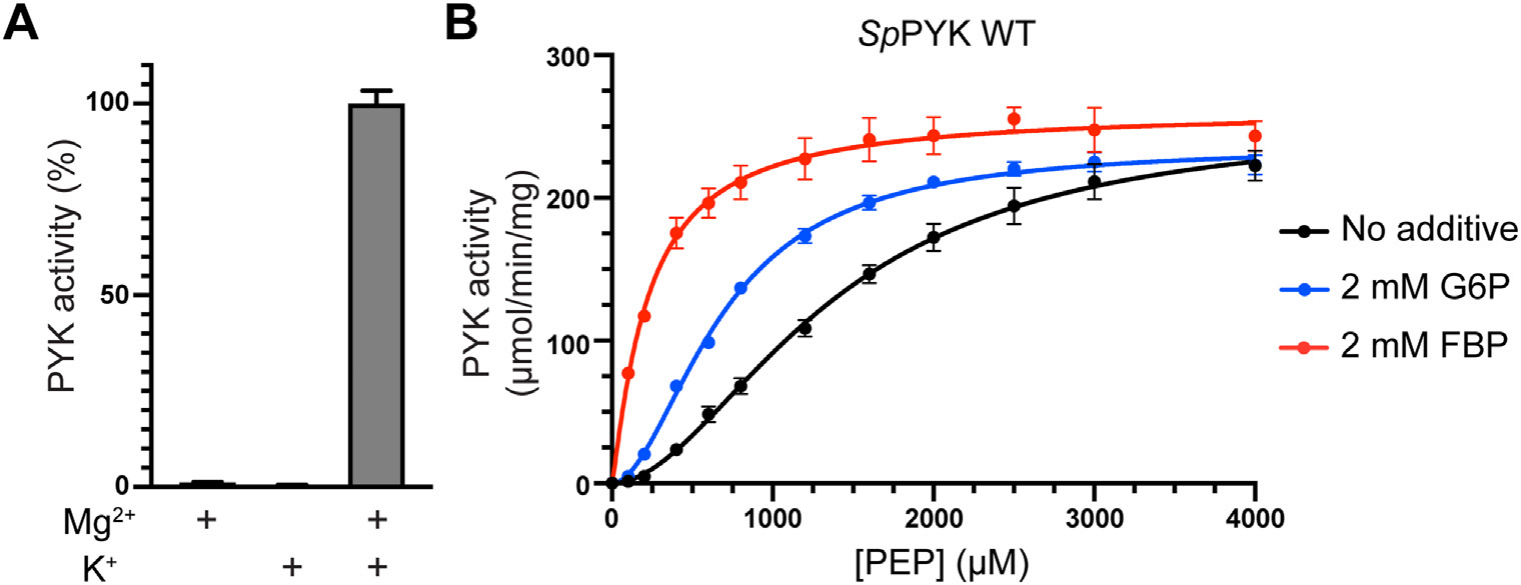
***Sp*PYK is activated by FBP and requires both Mg**^**2+**^**and K**^**+**^**for its activity.***A*, cation requirement was determined by measuring *Sp*PYK in the presence of 10 mM MgCl_2_ and/or 100 mM KCl. *B*, kinetic characterization of WT *Sp*PYK with respect to PEP in the presence of the indicated effector candidates. Error bars represent mean ± SD from triplicates. See Table S2 for kinetic values.

Although PYK is a key enzyme in the glycolysis pathway and has been studied in other organisms, *Sp*PYK has remained uncharacterized; we therefore purified *Sp*PYK and its variants to biochemically define their activities (Fig. S3). PYKs require divalent cations for activity, and they are either K^+^-dependent or K^+^-independent depending on the active site residue being a Glu or Lys, respectively (28). This residue is Glu93 in *Sp*PYK, and we found that both Mg^2+^ and K^+^ are necessary for the proper function of *Sp*PYK (Fig. 3*A*). Titration experiments with PEP showed a sigmodal kinetic profile with a *S*_0.5_ value of 1.36 ± 0.06 mM and a Hill coefficient (*h*) of 1.88 ± 0.10 (Fig. 3*B* and Table S2). Sigmoidal kinetics was also observed with respect to ADP concentration (*S*_0.5_ = 1.09 ± 0.06 mM, *h* = 1.47 ± 0.07) (Fig. S4). The positive cooperativity observed here points to a homotropic regulation of *Sp*PYK by its substrates.

We proceeded to identify the effector for *Sp*PYK because PYKs are known to be allosterically regulated by sugar phosphates present in the glycolytic pathway (12). Fructose 1,6-bisphosphate (FBP) and glucose 6-phosphate (G6P) have been reported as candidate effectors in other streptococcal PYKs; we therefore investigated whether the addition of these sugar phosphates changes the kinetic profile of *Sp*PYK (29, 30). The addition of FBP rendered the kinetic profile to a hyperbolic curve with a concomitant increase in PEP affinity as indicated from the lower *S*_0.5_ value (*S*_0.5_ = 0.22± 0.01 mM, *h* = 1.16 ± 0.09). In contrast, only a modest activation effect was observed upon G6P addition, and the kinetic profile remained sigmoidal (Fig. 3*B* and Table S2). These results demonstrate that FBP is the primary allosteric activator of *Sp*PYK. Next, we tested the *in vitro* activity of *Sp*PYK(A218V) and *Sp*PYK(T407A), which are mutants found in the fosfomycin-resistant strains that were predicted to have compromised activity from cellular studies. While the activation effect of FBP was still present in these variants, catalytic efficiency inferred from *k*_cat_/*S*_0.5_ values showed a ∼300-fold and ∼100-fold decrease in A218V and T407A, respectively, confirming the notion that the delayed growth phenotype is associated with the decrease in PYK activity (Fig. S5 and Table S2).

### Crystal structures of *Sp*PYK reveal inter-protomer interactions involved in allosteric regulation

**Figure 4:**
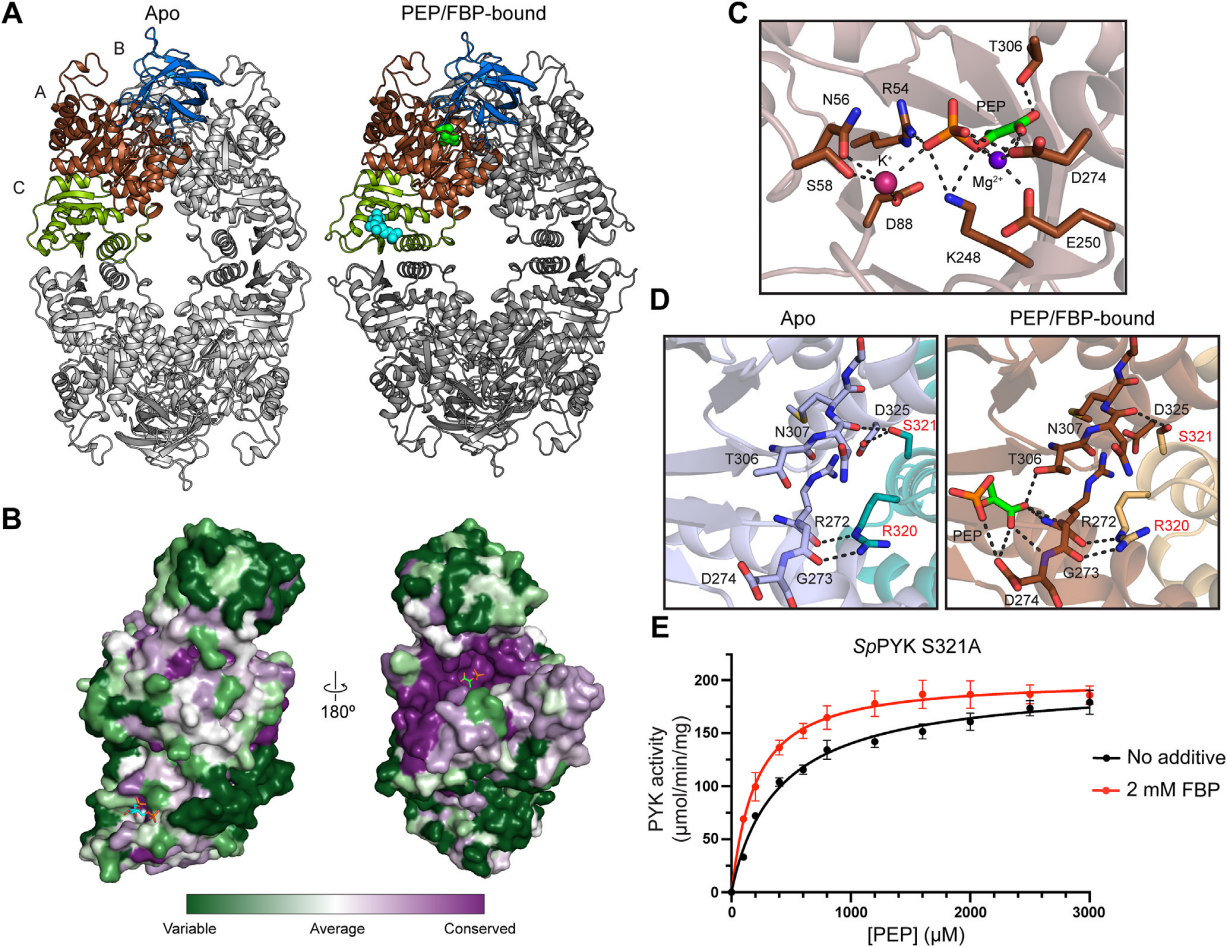
**Crystal structures of *Sp*PYK in apo and PEP/FBP-bound conformations highlight key residues at the catalytic site and A-A interface that are involved in coordinating catalytic activity.***A*, crystal structures of apo (*left*) and PEP/FBP-bound (*right*) *Sp*PYK. The *brown, blue, and light green* regions in the *top-left* protomer correspond to the *A*–*C* domains, respectively. PEP (*green*) and FBP (*cyan*) are only shown in the top-left protomer for clarity. *B*, the surface of PEP/FBP-bound *Sp*PYK protomer is colored by sequence conservation computed by the ConSurf Server (57). The loop between Aβ1 strand and Aα1 helix was omitted from the analysis. *C*, a close-up view of the *Sp*PYK active site shows the side chains of conserved residues that coordinate the binding of PEP and cations. *D*, Arg320 and Ser321 that are present in the A domain of the adjacent protomer (*red label*) interact with residues that constitute the active site (*black label*). The Ser321-Asp325 interaction is only seen in the FBP-free structures. *E*, the S321A variant displays a hyperbolic kinetic curve in the absence of FBP. Error bars represent mean ± SD from triplicates. See Table S2 for kinetic values.

Having established the biochemical properties of *Sp*PYK, we next focused on structurally characterizing *Sp*PYK to gain a better understanding of how its activity is modulated by the effector. Although PYK structures in complex with FBP are available from yeast and human PYKs, no analogous structure has been reported for prokaryotic PYKs (31, 32). To obtain *Sp*PYK structures in different conformations, we conducted vapor diffusion crystallization trials with FBP and PEP as well as the enolpyruvate analog oxalate that has previously been used for PYK crystallization studies (33, 34). We successfully obtained *Sp*PYK crystal structures in all ligand combinations including apo-*Sp*PYK (2.0 Å resolution) and PEP/FBP-bound *Sp*PYK (1.8 Å resolution), which provide insight into its conformational movement upon ligand binding (Table S3). The overall structure of *Sp*PYK, which consists of four protomers with three domains, shares the structural organization of archetypal PYKs (Fig. 4*A*). The bound PEP or oxalate is seen in the catalytic site located at the pocket flanked by A and B domains, and FBP is found at the effector binding site in the C domain located ∼35 Å away from the catalytic site. Conserved residues are concentrated in the A domain, with the highest degree of conservation seen in the catalytic site and its surrounding areas (Figs. 4*B* and S6). Residues in the effector binding site are more variable, which reflects local adaptation to different PYK effectors in different species. While *Sp*PYK shares most of the domain features observed in other PYKs, it contains an extra loop in the A domain between Aβ1 strand and Aα1 helix that is not present in the majority of PYKs (Fig. S6). A “rocking motion” mechanism has previously been described for eukaryotic and prokaryotic PYKs that involves the rigid body rotation of A and C domains during the transition between different conformations (33, 34, 35). Comparison of the apo and PEP/FBP-bound structures revealed a similar transition for *Sp*PYK, where FBP binding triggers the rotation of each protomer by ∼7 degrees while the overall structure of the individual protomer remained largely unchanged except for the C domain effector binding site and the mobile B domain (Cα root mean square deviation (RMSD) of the individual AC domains = 0.33 Å) (Fig. S7).

Our substrate-bound structures provide the first detailed look into the active site of a prokaryotic PYK containing the physiologically relevant substrate PEP and cations required for catalytic activity. The PEP phosphate group interacts with Arg54 and Lys248, which are ideally positioned to facilitate the phosphoryl transfer reaction in coordination with Mg^2+^ and K^+^ (Fig. 4*C*) (36, 37). The active site residues that interact with the ligands are identical to those identified in *Trypanosoma brucei* PYK, which demonstrates the highly conserved nature of the active site among PYKs (38). The binding of PEP and cations did not significantly alter the active site structure, which led us to wonder whether inter-protomer interactions between A domain residues are involved in the allosteric activation mechanism (Fig. S8). We selected Arg320 and Ser321 for further characterization because of their potential role in shaping the active site by stabilizing key residues that coordinate PEP binding (Fig. 4*D*). Strikingly, we obtained a hyperbolic kinetic profile for the S321A mutant in the absence of FBP, indicative of a loss in positive cooperativity (Fig. 4*E* and Table S2). In the apo-state, Ser321 forms a hydrogen bond with the universally conserved Asp325, and our result indicates that this interaction could be crucial in maintaining the inactive state of the enzyme in the absence of FBP as it was not present in any of the FBP-bound structures (Fig. S9). Reduced positive cooperativity was also observed for the R320A mutant, and both mutants only showed a modest increase in catalytic efficiency upon the addition of FBP (Fig. S10 and Table S2). These observations suggest that S321A and R320A mutations destabilize the inactive conformation and predispose the enzyme to a higher affinity state even in the absence of FBP. We conclude that A domain residues that are part of the interprotomer interface proximal to the active site are involved in the allosteric activation mechanism.

**Figure 5:**
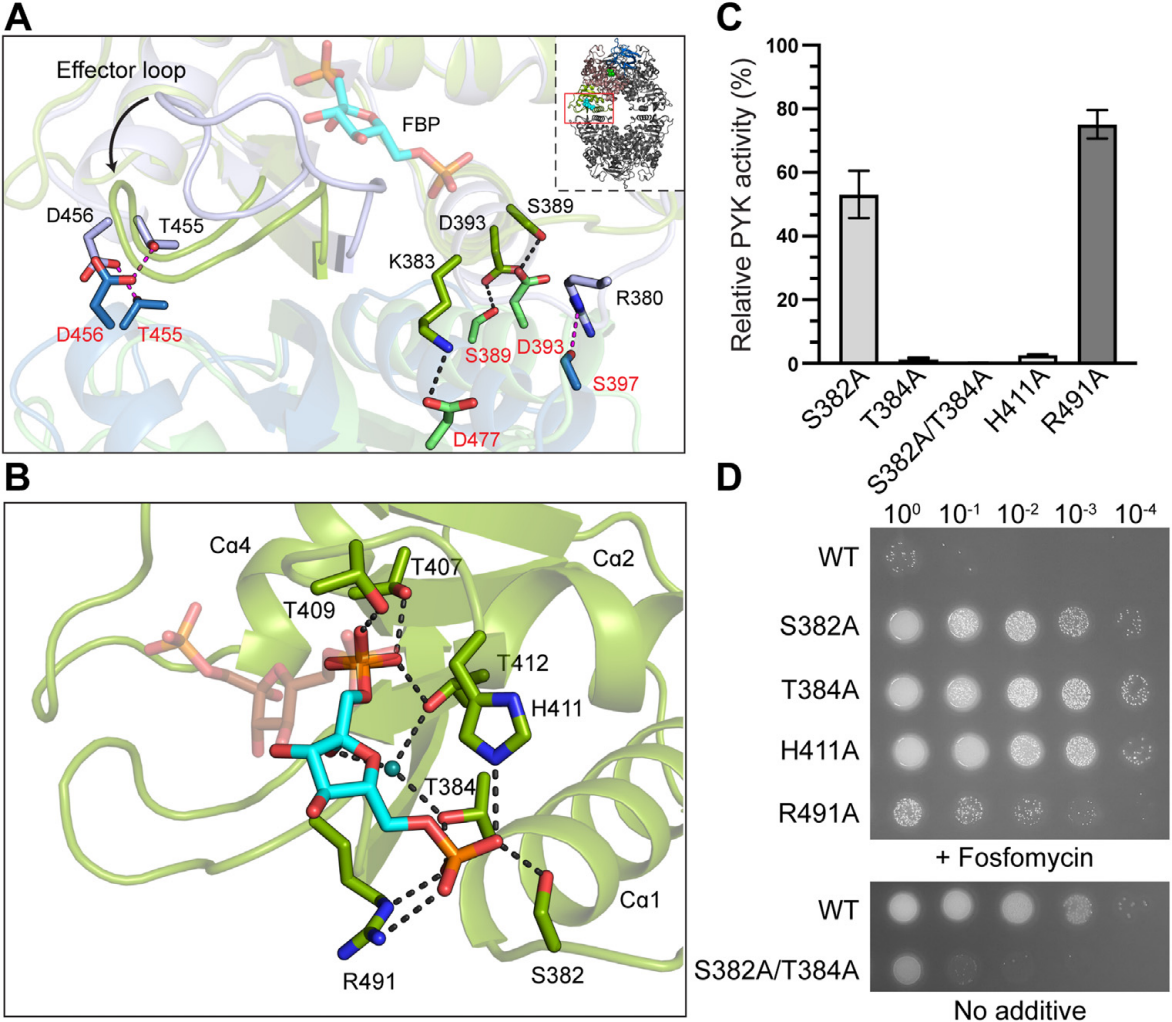
**FBP binding triggers a conformational reorganization at the C-C interface.***A*, overlay of apo (*light purple/blue*) and PEP/FBP-bound (*light green/green*) protomers at the C-C interface. Inter-protomer interactions seen in the apo structure (*magenta*) are disrupted by FBP binding, resulting in the formation of new interactions (*black*). *B*, side chains of effector binding site residues that interact with FBP (*cyan*) are shown. FBP observed in the human M2PYK structure (*brown*; Protein Data Bank: 4FXF) is overlayed for comparison. A water molecule observed in the effector binding site is shown in teal. *C*, *in vitro* PYK activity of the indicated alanine mutants relative to WT PYK was measured to assess the contribution of FBP interacting residues towards PYK activity in the presence of 3 mM ADP, 3 mM PEP, and 2 mM FBP. Error bars represent mean ± SD from triplicates. *D*, *S. pneumoniae* R6 cells with the indicated mutation at the native *pyk* locus were spotted on an agar plate containing fosfomycin (64 μg/ml) or an agar plate without any additives to assess viability.

The most notable structural reorganization upon effector binding occurs at the interface of the C domains (Fig. 5*A*). In the ligand-free structure, the side chains of Arg380-Ser397 and Thr455-Asp456 form hydrogen bonds that stabilize the interaction between the two protomers. Upon FBP binding, the effector loop (Ala482...Thr492) swings outward and destabilizes the Thr455-Asp456 interaction. This interface rearrangement is accompanied by the formation of new side-chain interactions including hydrogen bonds between Ser389 and Asp393. We identified a D393Y mutation in one of the fosfomycin-resistant isolates, which highlights the importance of this interaction in *Sp*PYK activity (Table S1). The C-C interface rearrangement was less pronounced in the PEP- or oxalate-only structure compared to the FBP-bound structures, suggesting a limited effect of substrate binding at the active site in inducing this conformational change (Fig. S11). Unexpectedly, we observed a weak yet clear electron density in the effector binding site of the oxalate-only structure that likely corresponds to a second oxalate molecule; whether this observation is physiologically relevant remains to be determined (Fig. S12).

### Effector binding residues are critical for *Sp*PYK activity and could be modified to recognize G6P

Although *Sp*PYK and human M2PYK both use FBP as their effector, the superposition of FBP-bound structures showed a marked difference in how FBP binding is coordinated (Figs. 5*B* and S13). In *Sp*PYK, the C1 phosphate is located at the effector site pocket and forms hydrogen bonds with semi-conserved threonine residues (Thr407, Thr409, and Thr412) located in the short loop region preceding the Cα2 helix. The fructose ring and the C6 phosphate point toward the C-C interface, with the latter making contacts with polar residues at the N-terminal end of the Cα1 helix (Ser382 and Thr384) as well as His411 in the short loop region and Arg491 on the effector loop. The fructose ring does not directly interact with *Sp*PYK residues; however, a water molecule is present in our crystal structure that is ideally positioned to form hydrogen bonds with the C2 hydroxyl of the sugar ring and threonine residues that also interact with the FBP phosphates, likely contributing to further stabilization of the protein-ligand interaction. In M2PYK, while the FBP C6 phosphate occupies the same effector site pocket as the FBP phosphate in *Sp*PYK, the rest of the FBP molecule is oriented away from the C-C interface, and the C1 phosphate interacts with residues on the Cα5 helix (Cα4 helix in *Sp*PYK) (32). Structural comparison of the C domain using available effector-bound PYK structures showed that the sugar ring of effector molecules in these structures overlaps with the FBP fructose ring in human M2PYK, which makes the FBP binding mode observed in *Sp*PYK unique among PYKs that have been characterized to date (Fig. S14).

To investigate the contribution of *Sp*PYK residues that interact with the FBP C6 phosphate toward PYK activity, we performed an *in vitro* characterization of single alanine mutants in the presence of FBP. The catalytic function was impaired for all mutants tested, with the most significant disruption of activity observed for T384A and H411A mutants (Fig. 5*C*). To evaluate whether these mutations affect *Sp*PYK activity in cells, we constructed strains containing the desired mutation in the native *pyk* locus and plated them on fosfomycin-containing media. In agreement with the biochemical data, cells containing alanine mutations became resistant to fosfomycin (Fig. 5*D*). Furthermore, the replacement of both Ser382 and Thr384 to alanines abolished *Sp*PYK activity *in vitro*, and cells solely expressing the double mutant were inviable (Fig. 5, *C* and *D*). Our genetic and biochemical data emphasize the critical role of effector-binding residues identified in the FBP-bound structures in mediating PYK activity.

**Figure 6:**
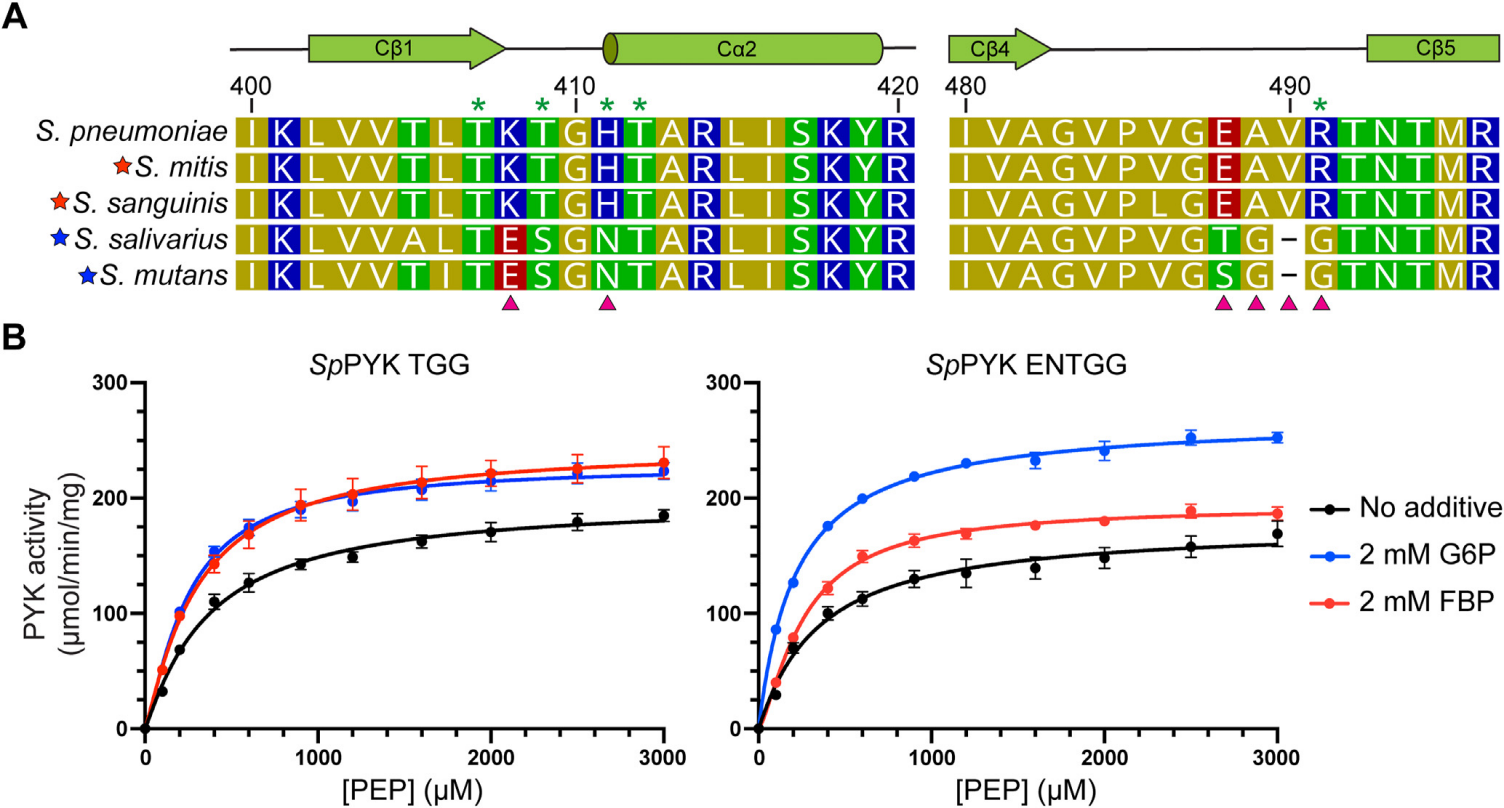
**Replacement of effector binding residues could alter *Sp*PYK effector preference.***A*, sequence alignment of streptococcal PYK residues around the effector binding site. *Green asterisks* denote residues that directly interact with FBP. Residues replaced to investigate effector selectivity are indicated by *red arrows*. *Red* and *blue stars* correspond to PYKs that have previously been reported to be activated by FBP and G6P, respectively (29, 30). See Fig. S15 for the complete sequence alignment of the C domain. *B*, kinetic curves for engineered *Sp*PYK with respect to PEP. TGG variant is responsive to both G6P and FBP while ENTGG variant is more responsive to G6P. Error bars represent mean ± SD from triplicates. See Table S2 for kinetic values.

The identification of the FBP binding site in *Sp*PYK prompted us to take a closer look at the effector binding sites of streptococcal PYKs activated by FBP or G6P. Sequence alignment of C domains revealed two regions containing FBP binding residues that are different between the two classes of PYKs (Figs. 6*A* and S15). The first region is located in the effector loop before Cβ5, where the Glu488...Arg491 residues in *Sp*PYK are replaced by TGG or SGG in G6P-activated PYKs. We found that replacing these residues in *Sp*PYK (*Sp*PYK TGG) converted the enzyme to be activated by both FBP and G6P (Fig. 6*B*). In the second region proximal to the semi-conserved threonine residues, the G6P-activated PYKs contain glutamate at the Lys408 position as well as an asparagine at the His411 position (Figs. 6*A* and S15). When these substitutions were introduced to *Sp*PYK TGG (*Sp*PYK ENTGG), the modified enzyme displayed a clear preference toward G6P over FBP for its activation and maintained wild-type activity levels (Fig. 6*B* and Table S2). Although the activity was moderately compromised, we observed a similar activator preference in the K408E/H411N mutant, suggesting that these two mutations confer G6P selectivity (Fig. S16). Our results demonstrate that the primary activator of streptococcal PYKs could be predicted from select effector binding site residues, and substitution of these residues could alter effector preference in *Sp*PYK.

## Discussion

In this work, we identified *S. pneumoniae* PYK mutants with increased fosfomycin resistance, which allowed us to gain insight into how these cells withstand the lethal effect of this antibiotic. PYK mutants are less efficient at converting PEP to pyruvate, and we have also shown that *S. pneumoniae* cells with lower PYK expression are less sensitive to fosfomycin. Our results are compatible with the resistance mechanism model where increased PEP availability due to deficient PYK activity leads to the native substrate outcompeting fosfomycin for the MurA active site in these cells. PYK point mutations have recently been reported in fosfomycin-resistant *Enterococcus faecium* and *Streptococcus thermophilus* strains obtained in a laboratory environment, suggesting that this mode of resistance could be widespread in streptococcal and enterococcal species (39, 40). It should be noted that mutations resulting in *in vitro* resistance does not necessarily reflect those seen *in vivo*. Indeed, clinical resistance to fosfomycin remained low in *E. coli* despite its rapid development of resistance *in vitro*, and this was attributed to the reduced growth rate of most resistant strains (41). We anticipate that the reduced fitness of *Sp*PYK mutants, which are unable to produce normal levels of pyruvate and its downstream metabolites, would prevent their growth in the host environment. Alanine is one of the key metabolites synthesized from pyruvate, and we speculate that DltD mutations observed in D39 PYK mutants are beneficial for growth by preventing alanine export to the cell surface and redirecting it to essential cellular processes (6, 26). Fosfomycin and other cell wall–targeting antibiotics have a synergistic effect in treating *S. pneumoniae* infections in animal models, and further studies on how resistance emerges *in vivo* may guide the development of effective treatments that utilize fosfomycin against multidrug-resistant *S. pneumoniae* (21).

The serendipitous isolation of PYK mutants led us to investigate the functional properties of *Sp*PYK, and we succeeded in solving the crystal structures of *Sp*PYK in various conformations, offering a first glance into the allosteric regulation of a prokaryotic PYK by FBP. The overall conformational change triggered by effector binding, which involves the structural reorganization of the C-C interface and protomer rotation, is universal among the allosterically regulated PYKs that have been characterized. However, how this change results in enhanced activity remains to be clarified because the structural alterations observed at the active site are subtle compared to the global domain movement. A side chain rotation of the conserved arginine located in the adjacent A domain has been implicated in stabilizing the active state by forming hydrogen bonds with the short alpha helix that interacts with PEP in human and *Mycobacterium tuberculosis* PYKs (33, 34). Our mutagenesis studies on the corresponding Arg320 in *Sp*PYK as well as the adjacent serine show that these residues participate in regulating activity. The drastic change in the kinetic profile of S321A mutant was especially notable because this residue is an alanine in all PYKs except for streptococcal PYKs (Fig. S6). Ser321 forms a hydrogen bond with Asp325 in the absence of FBP in *Sp*PYK, whereas this aspartate interacts with the conserved arginine in the apo-state of *M. tuberculosis* PYK (33). We presume that these interactions are required for the respective PYKs to maintain their inactive states in the absence of the effector. The importance of Ser321 in *Sp*PYK activity highlights a regulatory mechanism that is distinct from other characterized PYKs at the A-A interface, and tools such as molecular dynamics simulations may be used to elucidate this mechanism and gain a more comprehensive understanding of how allosteric activation is achieved (42, 43).

Our ligand-bound crystal structures provide an answer to a long-standing question that involves the coordination of FBP in prokaryotic PYKs. In human M2PYK, Trp482 and Arg489 on the Cα5 helix (Cα4 helix in *Sp*PYK) have been found to interact with the FBP C1 phosphate, and these residues are also involved in FBP binding in yeast PYK (31, 32). However, this helix is shorter in *Sp*PYK and *E. coli* PykF, and no residues on this helix are suitably positioned to coordinate FBP binding (44, 45) (Fig. S6). We show that the FBP C6 phosphate in *Sp*PYK does not occupy the same space as the FBP C1 phosphate in M2PYK, but instead positions itself where it interacts with polar residues close to the C-C interface. Polar residues that could mediate FBP binding are present in the same region in *E. coli*, and it is reasonable to speculate that *E. coli* PykF and other FBP-activated prokaryotic PYKs that share these features bind FBP similarly to *Sp*PYK (45) (Fig. S6).

We also identified residues that are important in the selective recognition of effector molecules by comparing the streptococcal PYK sequences and demonstrated that a G6P-activated *Sp*PYK could be engineered by modifying these residues. In the G6P-bound *P. aeruginosa* PykA structure, the glucose ring forms interactions with a serine on the effector loop and a glutamate in the effector site pocket (9). The threonine residue introduced in the effector loop in the TGG mutant may play a similar role by providing a binding site for G6P, which could in turn activate the mutant enzyme. A notable shift in selectivity towards G6P was observed for variants containing the K408E/H411N double mutation, which is not surprising given the importance of His411 in FBP recognition. The ability to design such mutants indicates that *Sp*PYK retains some degree of plasticity that allows it to maintain the active state even in the presumed absence of direct Cα1-effector interactions.

This study has conclusively established FBP as the primary activator of *Sp*PYK, but it does not exclude the existence of other regulatory mechanisms. In fact, several observations made in this study indicate that effector binding site residues in *Sp*PYK may possess a role beyond FBP recognition. The T407A mutation, which is only predicted to disrupt the interaction between *Sp*PYK and FBP C1 phosphate, results in a considerably lower PYK activity compared to that of the wild-type enzyme in the absence of FBP, implying that this mutation directly affects catalytic function. Effector loop mutants displayed a hyperbolic kinetic profile in the absence of effectors even though residues substituted in these mutants are not predicted to be directly involved in inter-protomer interaction. Considering that an oxalate molecule was observed in the effector binding site of the oxalate-only structure, it is possible that PYK substrates or products could regulate activity by interacting with effector binding site residues (Fig. S12). In addition, *Sp*PYK may contain noncanonical effector binding sites seen in other PYKs that provide an additional layer of allosteric regulation by binding to amino acids or sugar phosphates (23, 33, 46). These hypotheses could be addressed by employing genetic approaches combined with biochemical and structural validation.

This study now sets the stage for exploring *Sp*PYK inhibitors that could aid in antibiotic development. One challenge associated with targeting the glycolytic pathway for antibiotic development relates to the high degree of structural homology that is often seen between the prokaryotic and eukaryotic proteins and the need to discover bacterial selective inhibitors (47). Due to its high sequential and structural conservation, it is difficult to identify compounds that selectively bind to the active site cleft of prokaryotic PYKs. On the other hand, there are enough structural variabilities in the C domain containing the effector binding site and the protomer interface that could be exploited by small molecules designed to selectively prevent effector binding or conformational reorganization of the prokaryotic PYKs, and we have shown that even a single amino acid substitution in the C domain can greatly reduce *Sp*PYK activity. In *Staphylococcus aureus*, bis-indole alkaloid compounds have been reported to bind to the PYK C-C interface distal from the canonical effector site and possess antimicrobial properties by inhibiting PYK activity (48). While the spectrum of these compounds remains to be characterized, the discovery of bacterial PYK-specific inhibitors raises hope that *Sp*PYK inhibitors can be identified and optimized for use against multidrug-resistant *S. pneumoniae.*

## Experimental procedures

### Materials and bacterial culture conditions

*E. coli* strains were grown with shaking at 37 °C in lysogeny broth (LB) or on agarized LB plates with appropriate additives. *S. pneumoniae* strains were cultured statically in Todd Hewitt broth containing 0.5% yeast extract (THY) at 37 °C in an atmosphere containing 5% CO_2_. Pre-poured Trypticase Soy Agar with 5% Sheep Blood (TSAII 5% SB) plates with a 5 ml overlay of 1% nutrient broth agar or TSA plates containing 5% defibrinated sheep blood (Japan BioSerum) with appropriate additives were used for *S. pneumoniae* when growth on solid media was required. The following concentration of antibiotics was used: carbenicillin, 50 μg/ml; erythromycin, 0.2 μg/ml; gentamicin, 100 μg/ml; kanamycin, 50 μg/ml (*E. coli*) or 250 μg/ml (*S. pneumoniae*); spectinomycin, 200 μg/ml. The bacterial strains, plasmids and oligonucleotide primers used in this study are summarized in Tables S4–S6. The protocol for plasmid construction can be found in the Supporting information.

### Isolation of fosfomycin-resistant mutants and whole-genome sequencing

Fosfomycin-resistant mutants were isolated by plating D39 or R6 cells in the mid-log phase onto TSAII 5% SB plates supplemented with fosfomycin. Genomic DNA was isolated using a DNeasy Blood and Tissue Kit (QIAGEN), and sequencing was performed on the Illumina NovaSeq 6000 platform at the Genome Information Research Center (Osaka University). Sequencing reads were analyzed using Geneious Prime (v.2022.1.1) to identify single nucleotide polymorphisms and indel mutations that were unique to the resistant mutants compared with the parental strains.

### *S. pneumoniae* strain construction

A previously reported protocol was used to transform *S. pneumoniae* R6 and its derivatives (49). In brief, cells at the mid-log phase were diluted to OD_600_ ∼0.03 in THY containing 1 mM CaCl_2_ and 0.2% bovine serum albumin, and competence was stimulated by adding 500 ng/ml competence-stimulating peptide (CSP-1; GenScript). After 15 min incubation, 200 ng DNA product was added to a 1 ml culture, and the resulting culture was grown for 1 h. For transformant selection, 100 μl culture was combined with 5 ml molten 1% nutrient broth agar supplemented with appropriate additives, and the mixture was poured onto a TSAII 5% SB plate. Transformants were recovered after overnight incubation. A previously published method was used to introduce the desired mutation at the native *pyk* locus (50). AT1023 containing *lacI* and IPTG-inducible *pyk* was first transformed with a DNA cassette that includes an antibiotic resistance marker and *B. subtilis sacB* flanked by ∼1 kb upstream and downstream regions of *pyk*. After antibiotic selection, a DNA cassette containing the desired *pyk* mutation was transformed, and transformants were selected with 10% sucrose. These steps were performed in the presence of 1 mM IPTG to facilitate ectopic *pyk* expression. Detailed protocols for strain construction can be found in the Supporting information.

### *S. pneumoniae* viability assay, MIC determination, and growth assessment

For determining viability on solid media, *S. pneumoniae* cells at mid-log phase were collected by centrifugation and normalized to OD_600_ = 1. The samples were diluted 10-fold four times, and 3 μl dilution sample was spotted onto TSA 5% SB plates containing the indicated additives. Plates were imaged after overnight incubation. Liquid MIC was determined by first preparing mid-log phase *S. pneumoniae* cells normalized to OD_600_ = 1. The normalized culture was diluted 1:500 in THY and 100 μl of the diluted culture was added to each well of a 96-well plate containing 100 μl THY with fosfomycin. The plate was incubated for 18 h and OD_600_ was measured using the Infinite M200 Pro plate reader (Tecan). For the growth assessment of *S. pneumoniae* strains, cultures grown to mid-log phase were diluted to OD_600_ = 0.01, and growth was monitored by measuring OD_600_ at the indicated time points.

### Expression and purification of *Sp*PYK

*E. coli* BL21(DE3) derivative ECOS Sonic cells containing the expression plasmid of interest was grown in 1 L LB supplemented with kanamycin at 37 °C with shaking until OD_600_ ∼0.4. The culture was cooled to 18 °C and protein expression was induced by adding 500 μM IPTG. Cells were harvested 18 h post-induction by centrifugation (4200*g*, 15 min, 4 °C) and the pellet was stored at −80 °C. All purification steps were performed at 4 °C. Cells were resuspended in 25 ml buffer A (50 mM HEPES pH 7.5, 500 mM NaCl, and 10% glycerol) supplemented with 0.25 mg/ml DNase and one tablet of cOmplete EDTA-free Protease Inhibitor Cocktail (Roche), and the sample was lysed with French press. Cell debris was removed by centrifugation (10,000*g*, 5 min, 4 °C), and the soluble fraction was collected by ultracentrifugation (100,000*g*, 30 min, 4 °C). The resulting supernatant was supplemented with 0.5 ml pre-equilibrated His60 Ni superflow resin (Takara Bio) and 20 mM imidazole and the resulting mixture was stirred for 30 min at 4 °C. The sample was then loaded onto a gravity column and washed with 30 ml buffer A containing 25 mM imidazole followed by 30 ml buffer A containing 50 mM imidazole. The protein was then eluted in 5 ml buffer A containing 300 mM imidazole. To prepare protein samples used in enzymatic assays, Econo-Pac 10DG column (Bio-Rad) equilibrated with buffer A was used to remove imidazole from the eluate, and the sample was concentrated to 3 mg/ml by centrifugal filtration. Samples were flash-frozen with liquid nitrogen and stored at −80 °C. To prepare protein samples for structural studies, the eluate was further purified by size exclusion chromatography on a Superdex 200 10/300 Gl column (Cytiva) equilibrated with buffer B (20 mM HEPES pH 7.5, 150 mM NaCl). Fractions containing the target protein were concentrated by centrifugal filtration, and the concentrated sample was immediately subjected to crystallization trials.

### Crystallography and data collection

Vapor diffusion crystallization trials were performed at 25 °C by mixing 15 to 20 mg/ml protein solution with an equal volume of reservoir solution. For obtaining the ligand-bound structures, KCl, MgCl_2,_ and ligand(s) stocks prepared in buffer B were added to the *Sp*PYK sample in buffer B (final buffer composition: 20 mM HEPES pH 7.5, 150 mM NaCl, 100 mM KCl, 20 mM MgCl_2_ and 5–20 mM ligand(s)) before mixing the protein sample with the reservoir solution. For obtaining the apo structure, *Sp*PYK sample in buffer B without any additives was used. Crystal growth was observed within 4 days, with diffraction-quality crystals harvested over the course of 1 to 4 weeks. For cryoprotection, crystals were soaked in reservoir solution containing 25% ethylene glycol (FBP, PEP and PEP/FBP), 20% PEG 400 (Apo and Oxalate) or 40% PEG 300 (Oxalate/FBP) as cryoprotectant for several minutes. The following reservoir solutions were used to obtain crystals for each structure: Apo — 100 mM sodium citrate pH 5.5 and 22% polyethylene glycol (PEG) 1000; Oxalate — 2% Tacsimate pH 6.0, 100 mM Bis-Tris pH 6.5 and 20% PEG 3350; Oxalate/FBP — 100 mM sodium acetate pH 4.2 and 33% PEG 300; FBP — 300 mM ammonium chloride and 18% PEG 3350; PEP — 50 mM HEPES pH 5.4, 2 M ammonium sulfate, 20 mM MgCl_2_, 2 mM CoCl_2_, and 1 mM spermine; PEP/FBP — 100 mM citric acid/BIS-TRIS propane (3.75 : 6.75) and 16% PEG 3350.

Each X-ray diffraction dataset was collected at beamline BL44XU (SPring-8), with a EIGER X 16M detector at 100K. The X-ray diffraction data were processed and scaled using XDS (51). The initial model of PYK was determined by the molecular replacement method using MOLREP in the CCP4 program suite (52, 53). The search model for MOLREP was generated by AlphaFold2 (54). The initial model was refined and rebuilt using REFMAC5 and Coot (55, 56). The data collection and refinement statistics are summarized in Table S3. Figures were prepared using PyMOL (v.2.5.3).

The ConSurf Server was used to compute the degree of residue conservation in Figure 4*B* (57). In brief, a search of 150 close homologous sequences in the UniRef90 database was carried out by HMMER and a multiple sequence alignment of *Sp*PYK to these sequences was constructed using MAFFT. Conservation scores were assigned based on the relative degree of conservation, and the resulting scores were visualized with PyMOL.

### *In vitro* measurement of *Sp*PYK activity

The protocol for assessing *Sp*PYK activity and kinetics was adapted from a previously published protocol with some modifications (33). All measurements except for those intended to identify cation dependence were performed at 25 °C in 100 μl reaction mixture containing 1x reaction buffer (20 mM HEPES pH 7.5, 100 mM KCl, 10 mM MgCl_2_, 0.6 mM NADH, 5 U lactate dehydrogenase) and *Sp*PYK. G6P (2 mM) or FBP (2 mM) was added when necessary. To identify cation dependence, 1 mM ethylenediaminetetraacetic acid (EDTA) was added to chelate residual divalent cation in the reaction mixture prior to the addition of indicated cations. For measuring enzyme kinetics with respect to ADP, PEP concentration was fixed at 3 mM and ADP was added to the reaction mixture to initiate the reaction. For measuring enzyme kinetics with respect to PEP, ADP concentration was fixed at 3 mM and PEP was added to the reaction mixture to initiate the reaction. *Sp*PYK activity was assessed by measuring the decrease in absorbance at 340 nm using an Infinite M200 Pro plate reader (Tecan). Kinetic analysis was performed with GraphPad Prism 9 (v.9.4.1) using an allosteric sigmoidal model.

## Data availability

X-ray crystallography data for *Sp*PYK have been deposited in Protein Data Bank under the accession numbers 8IAS (Apo), 8IAT (Oxalate), 8IAU (Oxalate/FBP), 8IAV (FBP), 8IAW (PEP) and 8IAX (PEP/FBP). All other study data are included within the manuscript.

## Supporting information

This article contains supporting information (58, 59, 60).

## Conflict of interest

The authors declare that they have no conflicts of interest with the contents of this article.
