# Supplementary material for: Functional and structural characterization of Streptococcus pneumoniae pyruvate kinase involved in fosfomycin resistance
Source: J Biol Chem. 2023 Jun 5;299(7):104892. doi: 10.1016/j.jbc.2023.104892 (PMC10338316; doi:10.1016/j.jbc.2023.104892)
Supplement: Supporting information [file mmc1.docx]

**SUPPORTING INFORMATION**

**Functional and structural characterization of *Streptococcus pneumoniae* pyruvate kinase involved in fosfomycin resistance**

Atsushi Taguchi^1,2*^, Ryosuke Nakashima^1^ and Kunihiko Nishino^1,2,3*^

^1^ SANKEN (The Institute of Scientific and Industrial Research), Osaka University, Ibaraki, Osaka 567-0047, Japan

^2^ Graduate School of Pharmaceutical Sciences, Osaka University, Suita, Osaka 565-0871, Japan

^3^ Center for Infectious Disease Education and Research, Osaka University, Suita, Osaka 565-0871, Japan

*email: taguchi@sanken.osaka-u.ac.jp; nishino@sanken.osaka-u.ac.jp

**Supplementary Methods**

**Plasmid construction**

His-*Sp*PYK expression in *E. coli*: The *pyk* (*SPD_0790*) gene was amplified from *S. pneumoniae* D39 genomic DNA using primers oAT201/oAT202. After digestion with NdeI and BamHI, the PCR product was ligated into pET28b. The resulting plasmid pATOS125 expresses His_6_-*Sp*PYK. Plasmids expressing mutant *Sp*PYK were constructed either by amplifying *pyk* from the genomic DNA of fosfomycin resistant isolates or introducing the desired mutation in pATOS125 with InFusion Cloning using the following primer pairs: S382A, oAT203/oAT204; T384A, oAT205/oAT206; H411A, oAT207/oAT208; R491A, oAT209/oAT210; S382AT384A, oAT211/oAT212; S321A, oAT213/oAT214; R320A, oAT215/oAT216; K408EH411N, oAT234/oAT235; E488_R491delinsTGG, oAT236/oAT237.

Ectopic *Sp*PYK expression in *S. pneumoniae*: The pPEPZ-P_lac_ vector was first PCR linearized using primers oAT217/oAT218. This linearized vector was ligated to *pyk* (oAT219/oAT220) via InFusion Cloning to generate pATOS133 (P_lac_-*pyk*).

***S. pneumoniae* strain construction**

AT1003 (*lacI*) and AT1023 (*lacI*, P_lac_-*pyk*): AT1003, which constitutively expresses *lacI*, was constructed by transforming pPEPY-PF6-lacI to *S. pneumoniae* R6 and selecting transformants with gentamicin. AT1023, which contains P_lac_-*pyk*, was constructed by transforming pATOS133 to AT1003 and selecting transformants with spectinomycin.

AT1275 (*lacI*, P_lac_-*pyk*, ∆*pyk::erm*, *sacB*), AT1285 (*lacI*, P_lac_-*pyk*, ∆*pyk*) and AT1405-1409 (*lacI*, P_lac_-*pyk* and *pyk* variants): The ~1kb upstream and downstream regions of *pyk* were amplified using primer pairs oAT221/oAT222 and oAT223/oAT224, respectively. Overlap extension PCR was used to assemble these PCR fragments with an antibiotic cassette containing *erm* and *sacB*, which was amplified using primers oAT225/oAT226. The resulting PCR product was transformed into AT1023 in the presence of 1 mM IPTG and selected with erythromycin to generate AT1275. For deleting the native *pyk* gene, the ~1kb upstream and downstream regions of *pyk* were amplified using oAT221/oAT227 and oAT224/oAT228 and ligated using overlap extension PCR. This deletion cassette was transformed into AT1275 in the presence of 1 mM and 10% sucrose to generate AT1285 that contains a clean *pyk* deletion. For constructing *pyk* point mutations at the native locus, primers oAT229/oAT230 were used to amplify *pyk* containing the desired mutation and the amplification product was ligated to *pyk* upstream and downstream regions (prepared with PCR primer pairs oAT221/oAT227 and oAT224/oAT231, respectively) using overlap extension PCR. The resulting PCR product was transformed into AT1275 in the presence of 1 mM IPTG and selected with 10% sucrose to generate AT1405-AT1409. The integration of PCR cassettes into the *pyk* locus was confirmed with diagnostic PCR using primers oAT232/oAT233 and DNA sequencing.


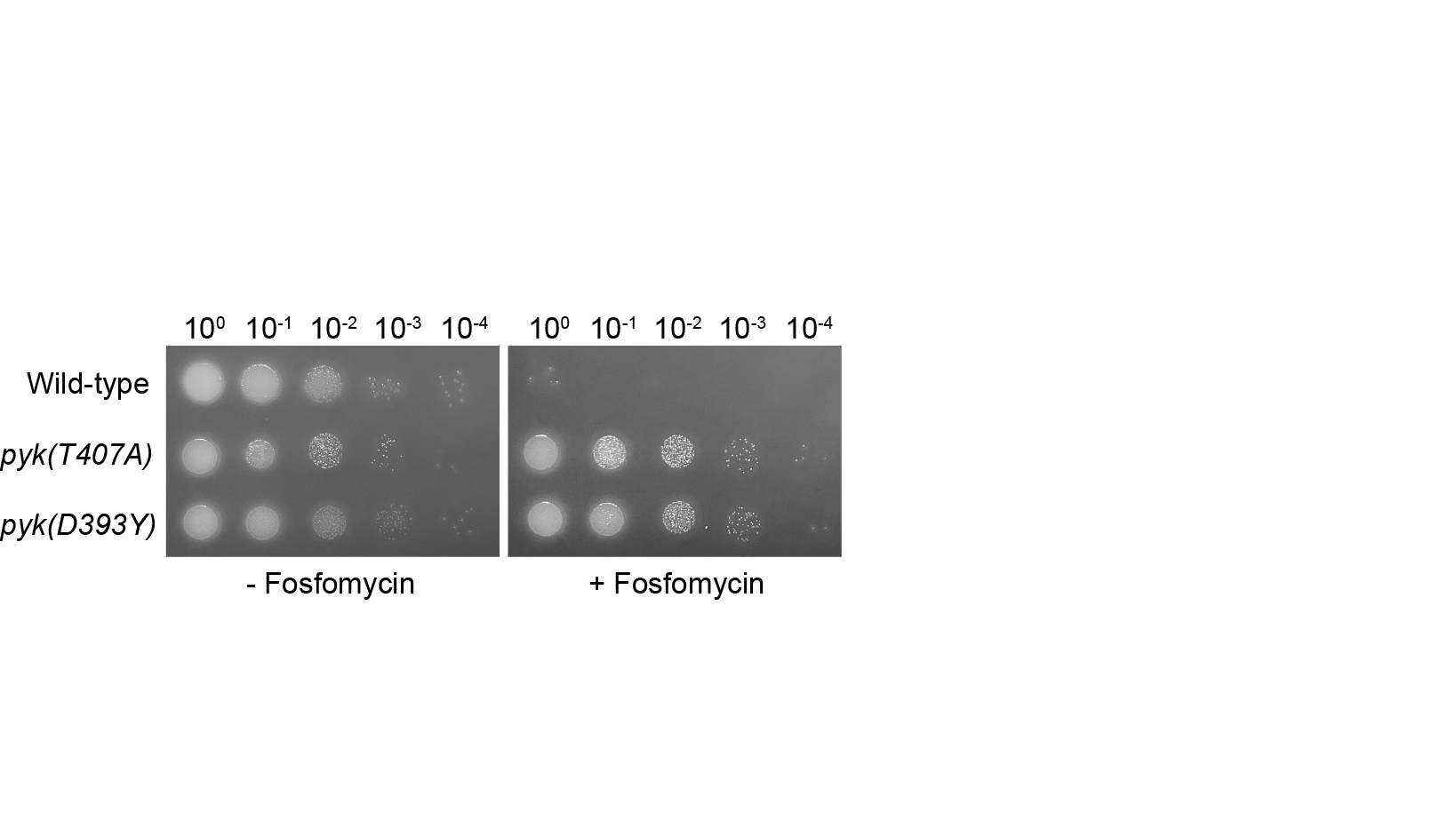


**Figure S1.** ***S. pneumoniae* R6 pyruvate kinase mutants can grow on solid media with lethal concentration of fosfomycin.** Wild-type and pyruvate kinase mutants were spotted on an agar plate containing fosfomycin (64 µg/mL) to evaluate resistance. Representative results from two independent experiments are shown.

**
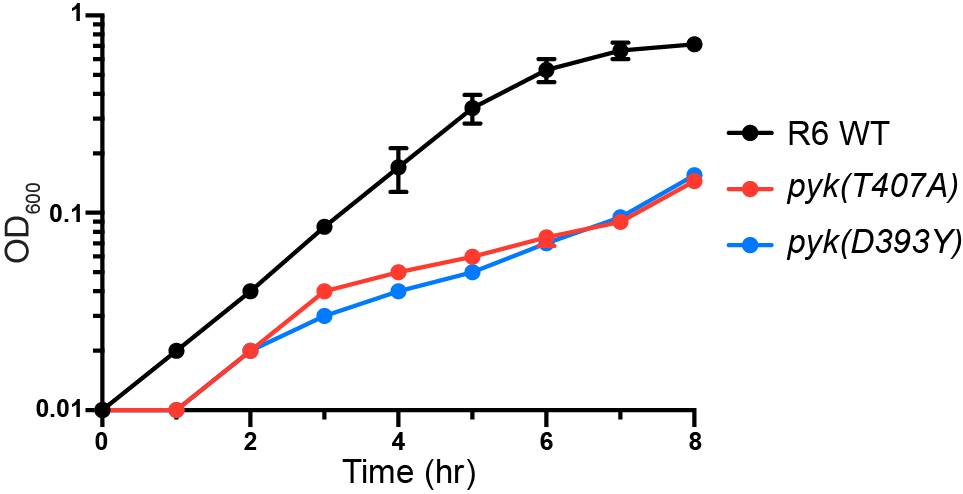
**

**Figure S2.** **Fosfomycin resistant mutants obtained from *S. pneumoniae* R6 strain show reduced growth rate in liquid culture.** Representative growth curves from three independent experiments are shown. Error bars represent mean ± SD from triplicates.

**
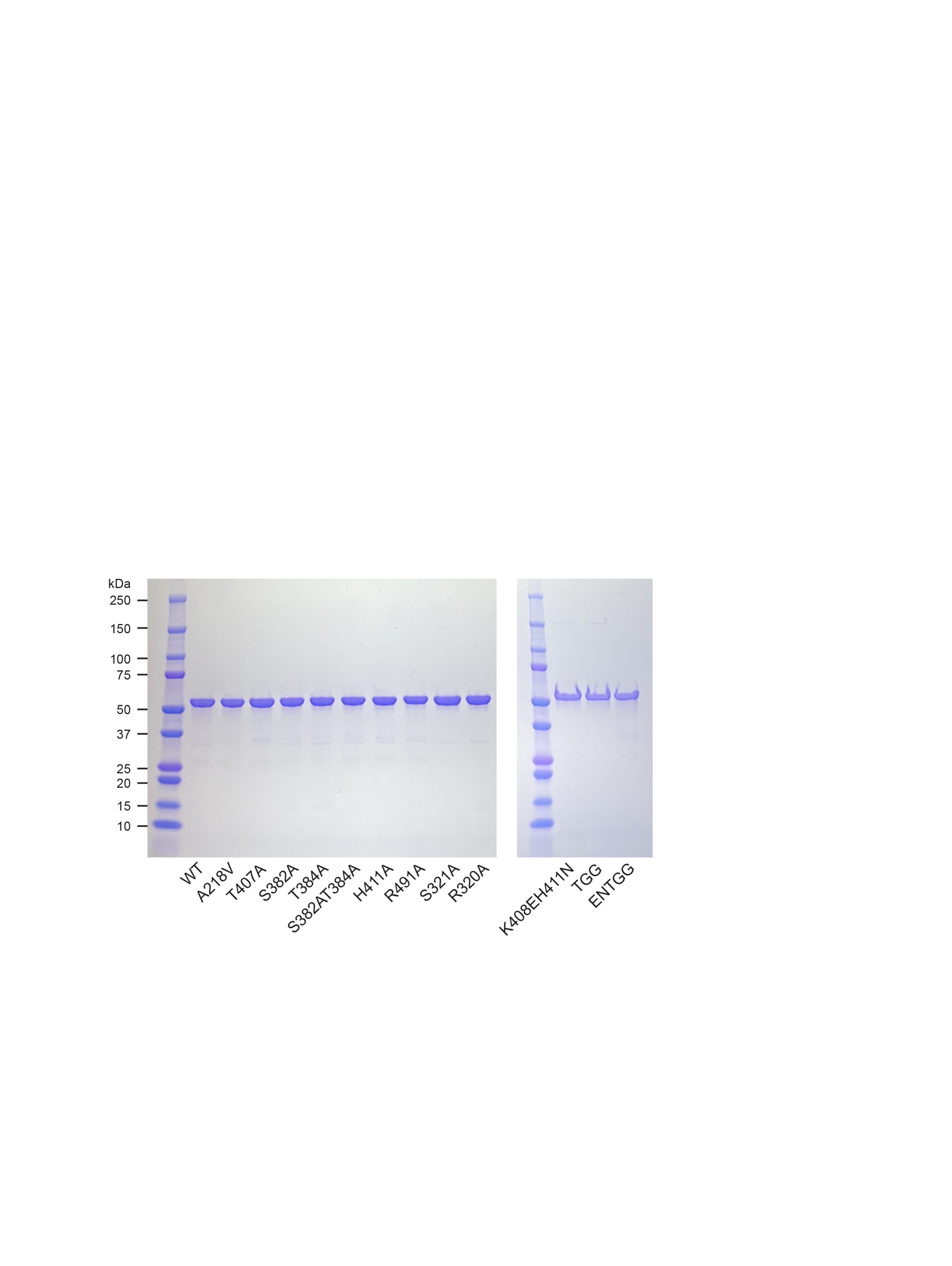
**

**Figure S3.** **Coomassie stained gel of purified *Sp*PYK used in this study.** ~2 µg protein was loaded per lane.


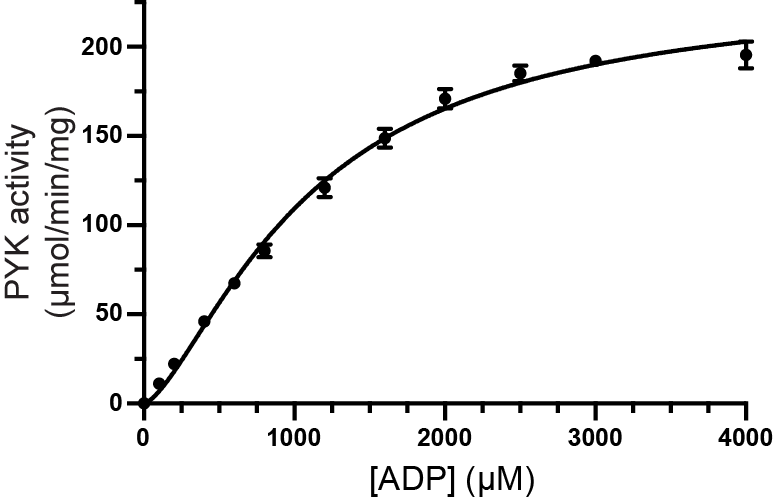


**Figure S4. Steady-state kinetics of WT *Sp*PYK under different ADP concentrations.** A representative kinetic curve from three independent experiments is shown. Error bars represent mean ± SD from triplicates. Kinetic values are as follows: *S*_0.5_ = 1.09 ± 0.06 mM; *h* = 1.47 ± 0.07; *k*_cat_ = 245.4 ± 7.7 s^-1^; *k*_cat_/*S*_0.5_ = 225.


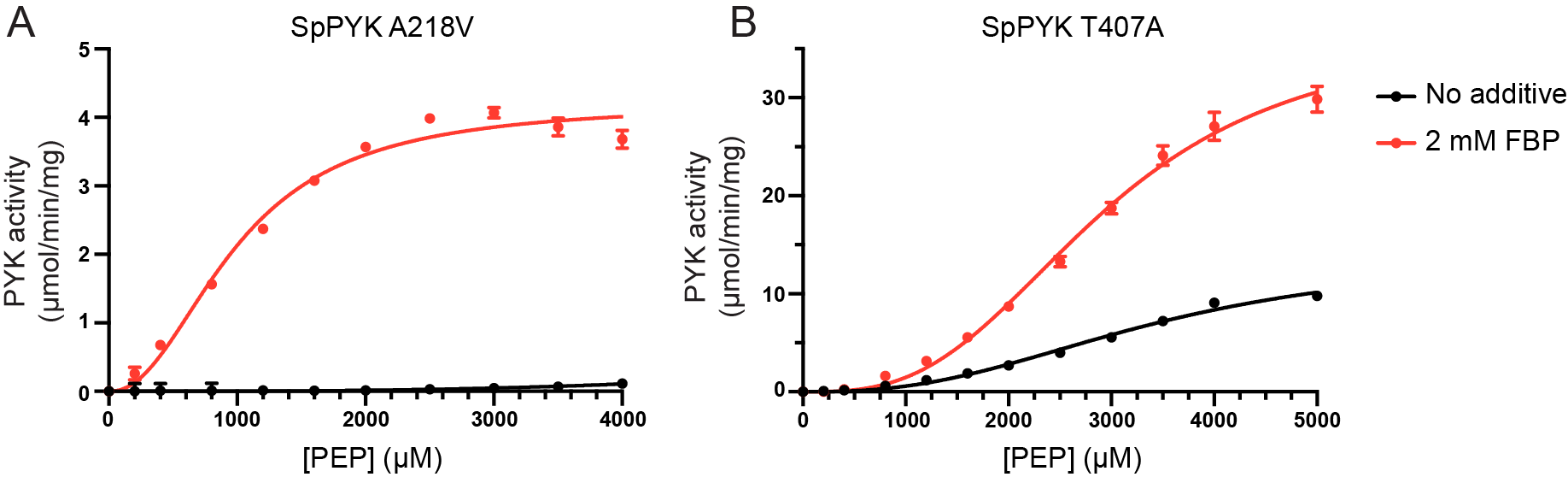


**Figure S5. *Sp*PYK(A218V) and *Sp*PYK(T407A) kinetics with respect to PEP.** Kinetic curves are shown for A218V (A) and T407A (B). Error bars represent mean ± SD from triplicates. See Table S2 for kinetic values.


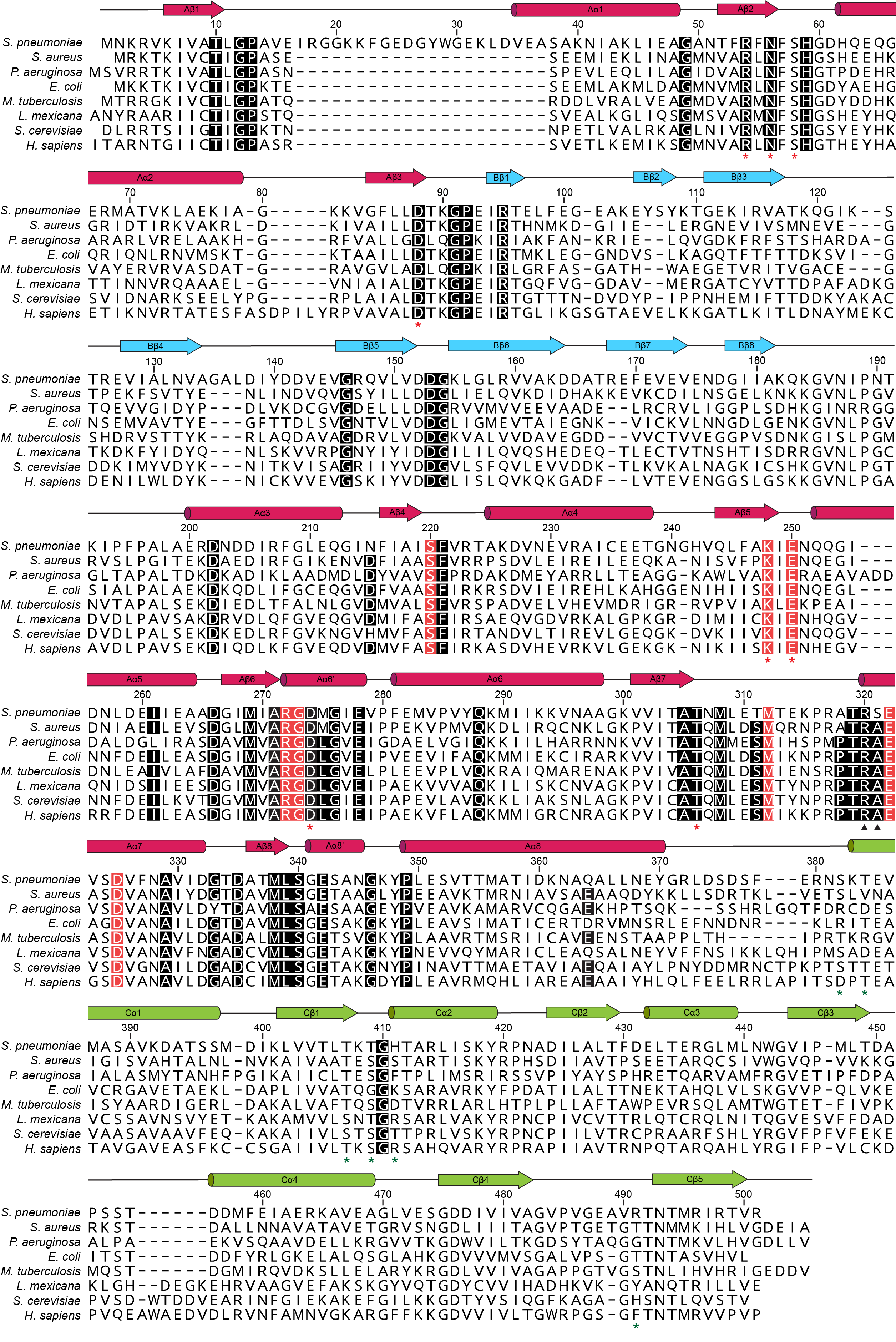


**Figure S6.** **Sequence alignment of PYK.** Sequence conservation analysis of ~19000 PYK sequences was performed using the EVcouplings server (58). Representative examples from 8 species (*Streptococcus pneumoniae*: SPD_0790; *Staphylococcus aureus*: SAOUHSC_01806; *Pseudomonas aeruginosa*: PA4329 (PykA); *Escherichia coli*: b1676 (PykF); *Mycobacterium tuberculosis*: Rv1617; *Leishmania mexicana*: LmxM.34.0030; *Saccharomyces cerevisiae*: YAL038W (PYK1); *Homo sapiens*: 5315 (PYM)) are shown. Residue numbers are based on the *Sp*PYK sequence, and secondary structure elements are shown based on the *Sp*PYK structure. The N domains in eukaryotic PYKs and the C' domain in *S. aureus* PYK have been omitted for clarity. Residues conserved in >90% and >99% of the analyzed sequences are highlighted in black and red, respectively. The red asterisks denote the residues found to interact with PEP and cations at the active site (Figure 4C). The green asterisks denote the residues found to interact with FBP at the effector binding site (Figure 5B). The black arrows indicate the residues at the A-A interface that were investigated in this study (Figure 4D).


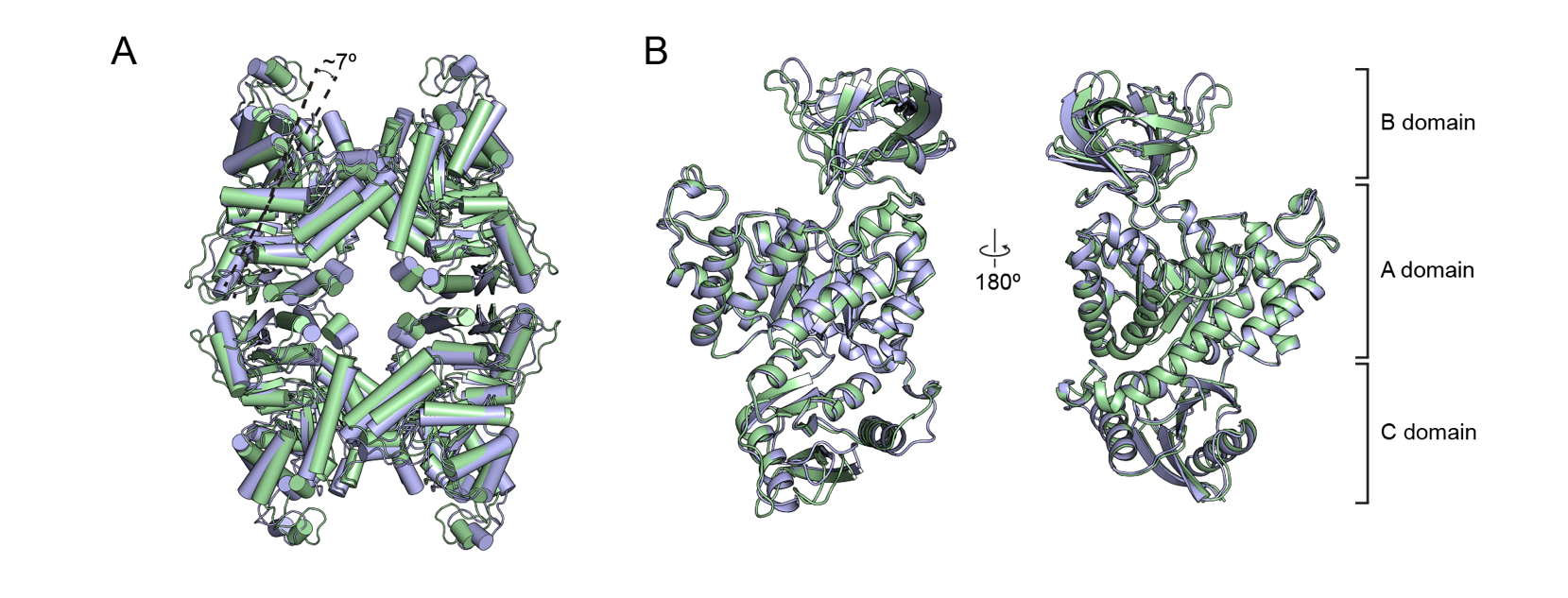


**Figure S7.** ***Sp*PYK undergoes a rigid-body rotation upon ligand binding.** (A) Overlay of the AC-domain of apo (light purple) and PEP/FBP-bound (pale green) structures. (B) Overlay of individual protomers from apo (light purple) and PEP/FBP-bound (pale green) structures.


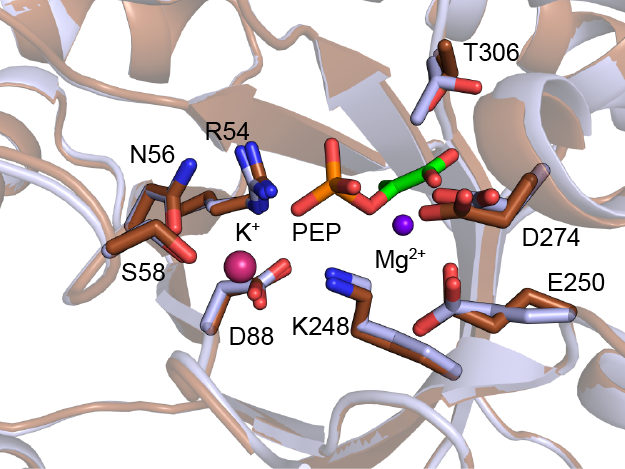


**Figure S8.** **No significant structural changes are observed in the active site upon ligand binding.** Overlay of the apo-structure active site (light purple) and PEP/FBP-bound active site (brown) is shown together with the PEP (green), K^+^ (red) and Mg^2+^ (purple) observed in the ligand-bound structure.


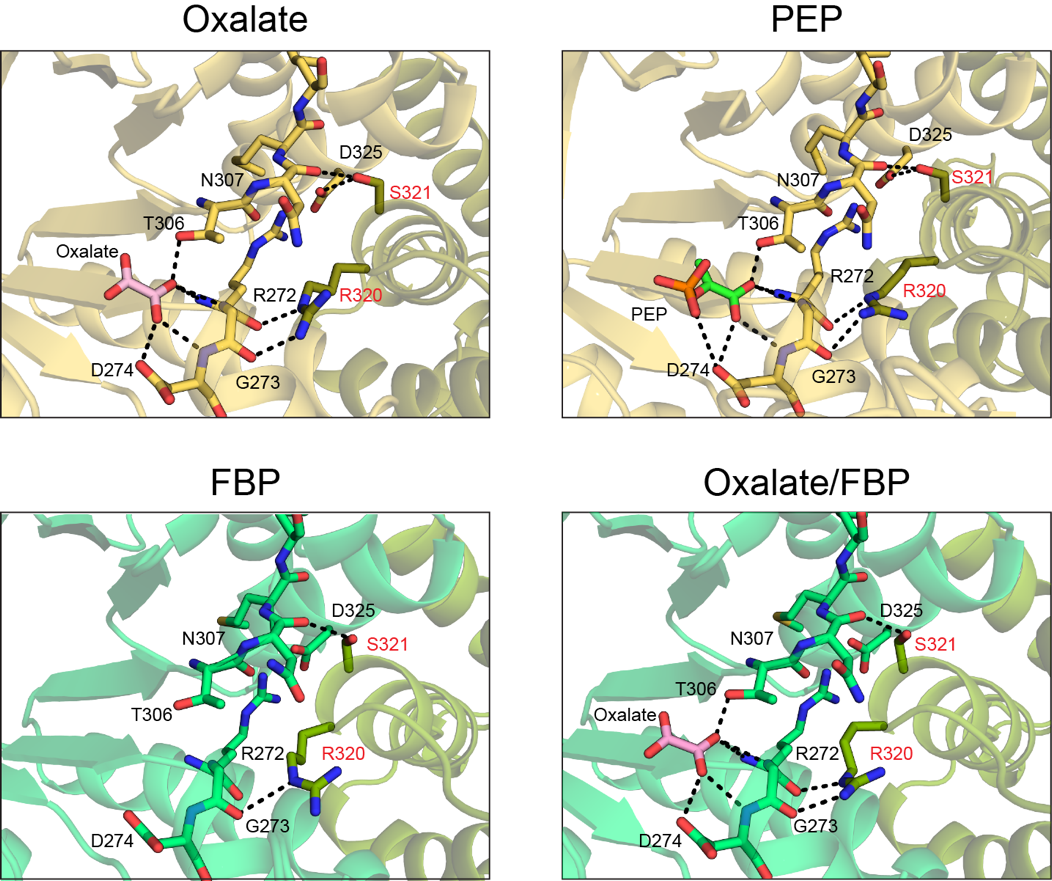


**Figure S9. FBP binding triggers a conformational change at the A-A interface.** The A-A interfaces of oxalate-, PEP-, FBP- and oxalate/FBP-bound structures are shown. Ser321 does not form a hydrogen bond with Asp325 in the FBP-bound structures.

**
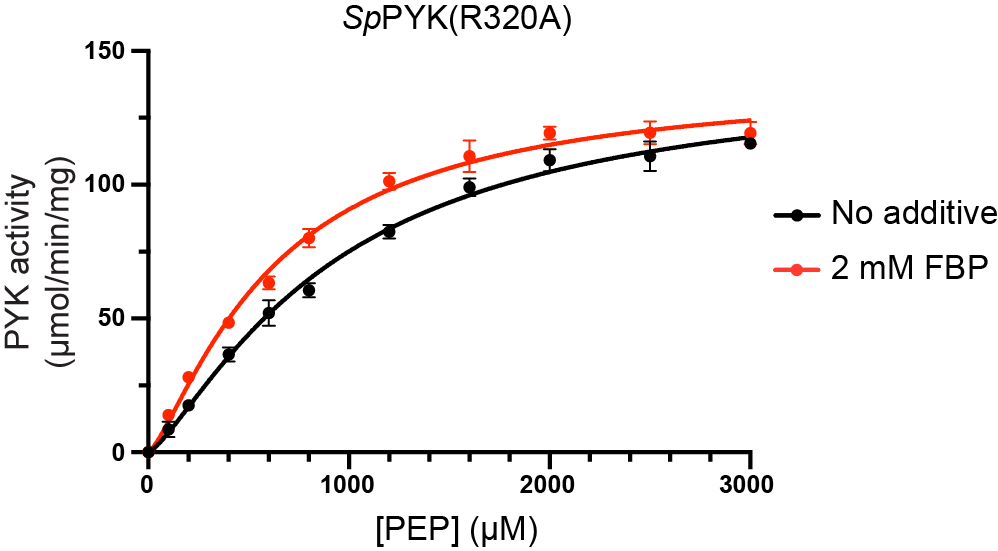
**

**Figure S10. *Sp*PYK(R320A) kinetics with respect to PEP.** Error bars represent mean ± SD from triplicates. See Table S2 for kinetic values.


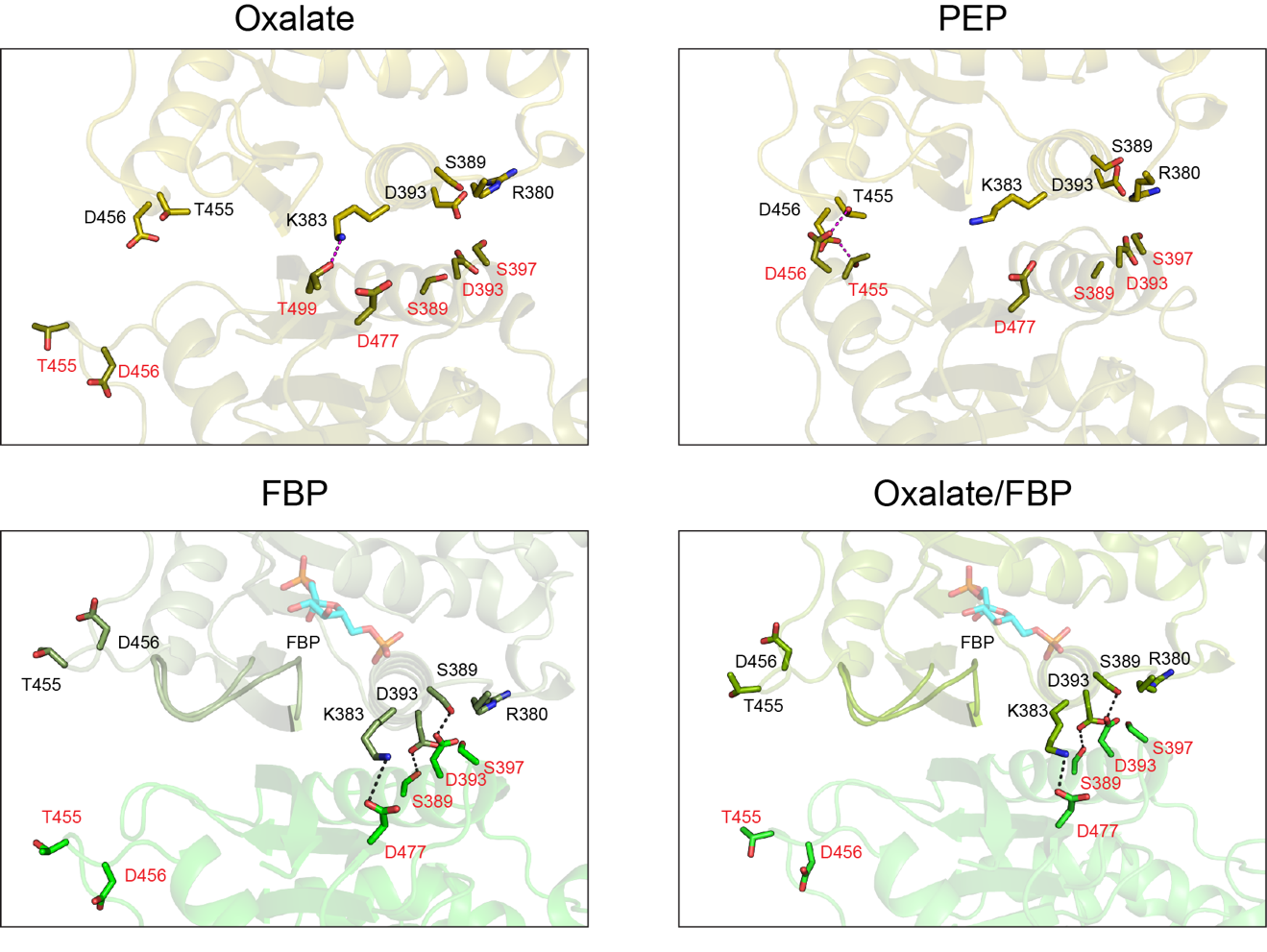


**Figure S11.** ***Sp*PYK structures without FBP have different C-C domain interactions compared to those containing FBP.** The C-C interfaces of oxalate-, PEP-, FBP- and oxalate/FBP-bound structures are shown. Only the FBP bound to the top protomer is displayed for clarity. Interactions between the Cβ5 residues are observed in all *Sp*PYK conformations in addition to the indicated interactions.


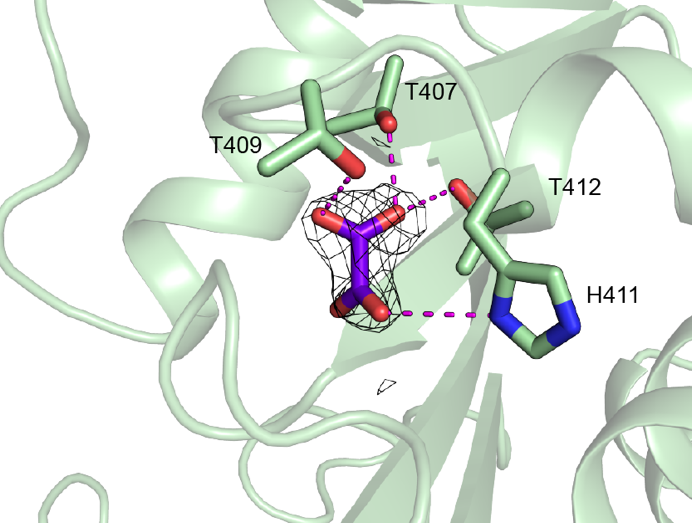


**Figure S12.** **Electron densities corresponding to oxalate are observed at the effector binding sites in the oxalate-only *Sp*PYK structure.** The 2Fo-Fc electron density (black mesh) for the ligand predicted to be an oxalate is contoured at 1σ. Pyruvic acid was the top prediction according to the automated ligand identification feature in the *PHENIX* software (59). Residues in the effector binding site that may interact with this ligand as well as their putative interactions are shown.


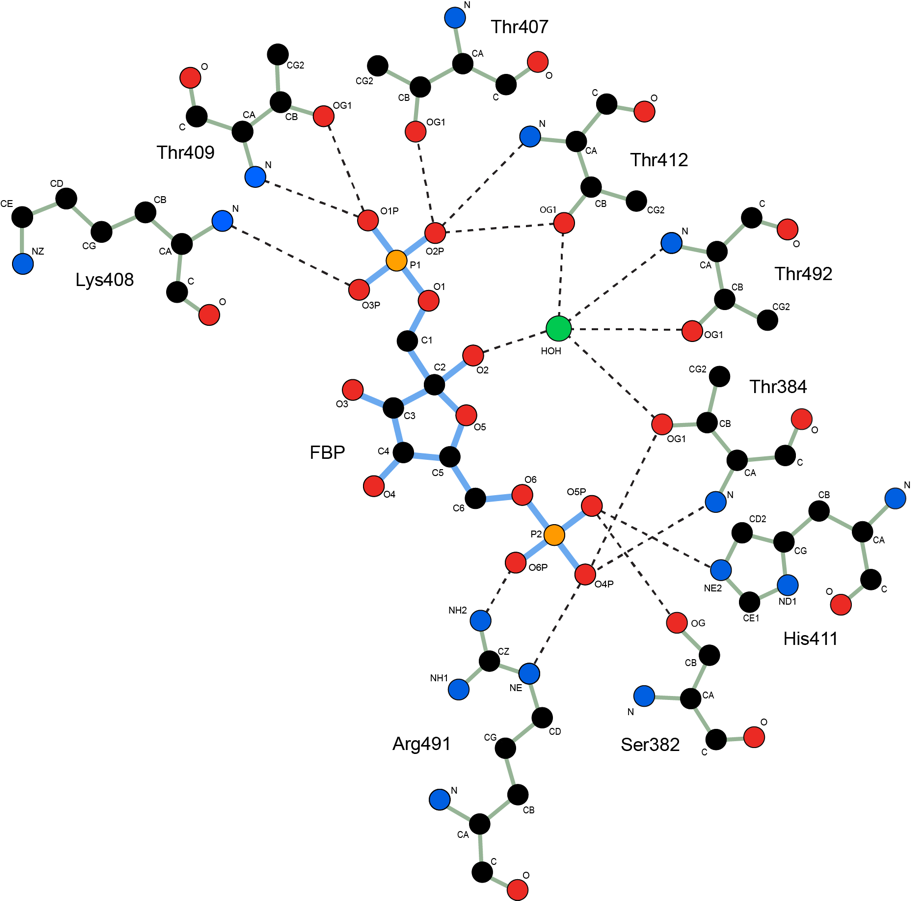


**Figure S13.** **Schematic showing the putative interactions between *Sp*PYK and FBP in the FBP/PEP-bound structure.** Carbon, oxygen, nitrogen and phosphate atoms are colored in black, red, blue and orange, respectively. A water molecule observed in the effector binding site that is part of the hydrogen bond network is shown in teal.


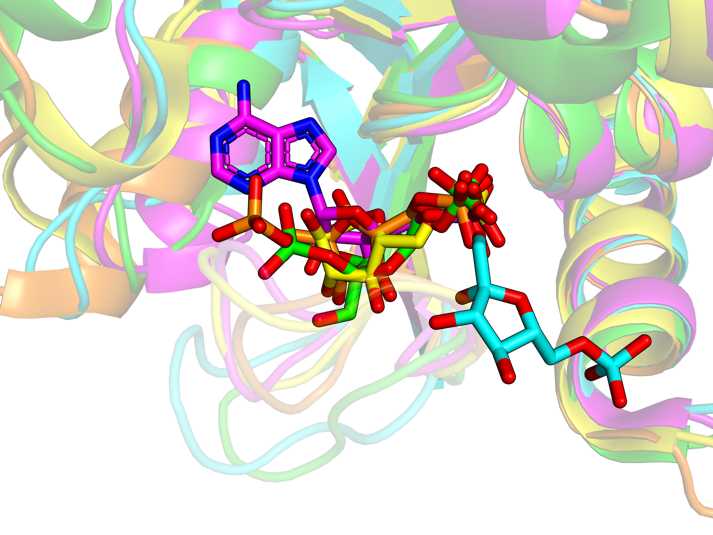


**Figure S14.** **Structural comparison of the effector binding domain from representative PYKs.** The overlayed effector binding domain structures of ligand-bound PYKs are shown.

Cyan: *S. pneumoniae* PYK with fructose 1,6-bisphoshate

Orange: *H. sapiens* M2PYK with fructose 1,6-bisphosphate (PDB: 4FXF)

Magenta: *M. tuberculosis* PYK with adenosine monophosphate (PDB: 5WSB)

Yellow: *P. aeruginosa* PykA with glucose 6-phosphate (PDB: 6QXL)

Green: *L. mexicana* PYK with fructose 2,6-bisphosphate (PDB: 3HQP)


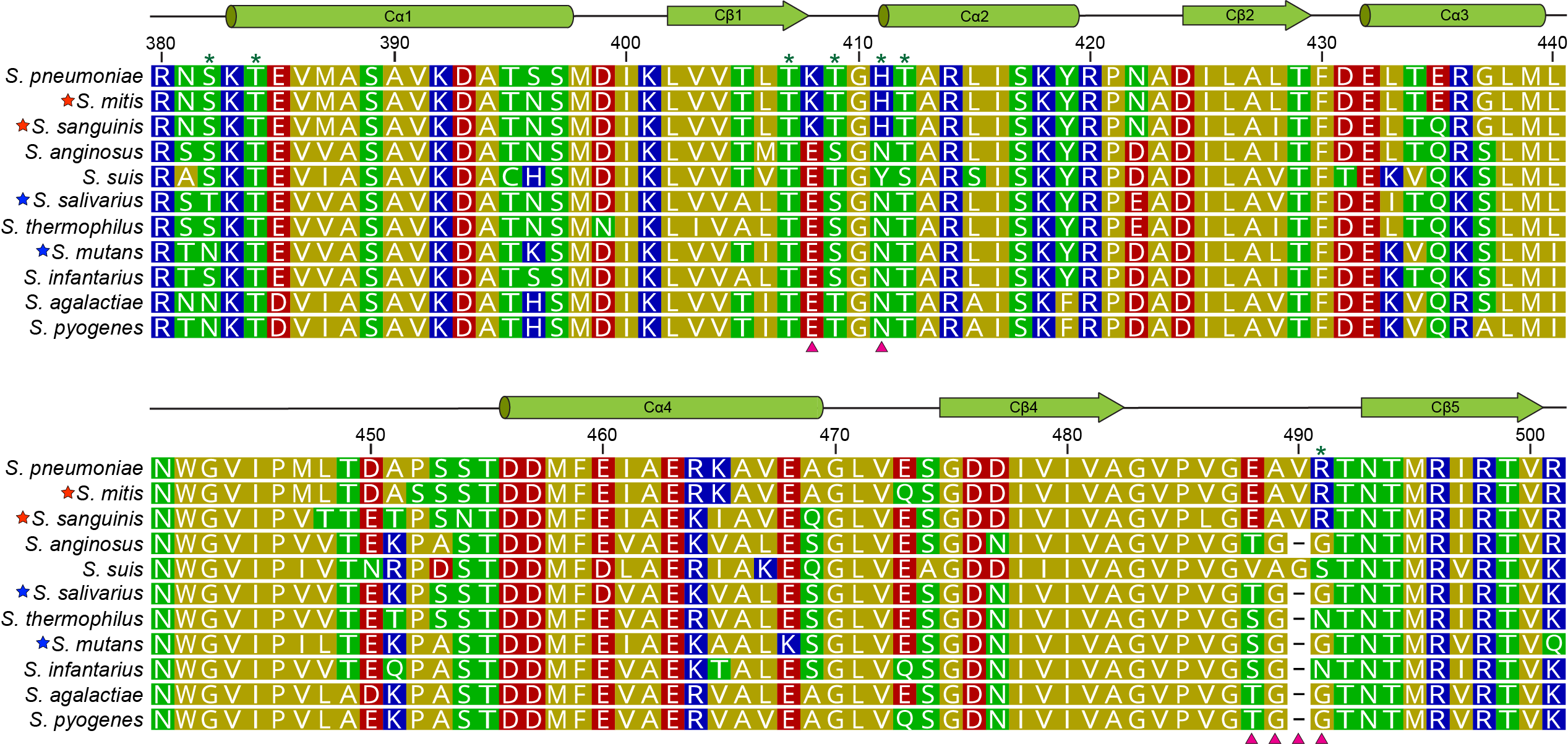


**Figure S15.** **Sequence alignment of *Streptococcus* PYK C domain.** Residue numbers are based on the *Sp*PYK sequence. Residues are colored based on polarity. The green asterisks denote the residues found to interact with FBP at the effector binding site (Figure 5B). The red arrows indicate residues investigated for G6P selectivity. Representative examples from 11 species (*Streptococcus pneumoniae*: SPD_0790; *Streptococcus mitis*: smi_1005; *Streptococcus sanguinis*: SSA_0848; *Streptococcus anginosus*: SAIN_1139; *Streptococcus suis*: SSUBM407_1319; *Streptococcus salivarius*: Ssal_01268; *Streptococcus thermophilus*: stu1196; *Streptococcus mutans*: SMU_1190; *Streptococcus infantarius*: Sinf_0755; *Streptococcus agalactiae*: gbs0931; *Streptococcus pyogenes*: SPy1282) are shown. FBP- and G6P-activated PYKs are indicated by red and blue stars, respectively, based on a previous report by Abbe and colleagues (29).


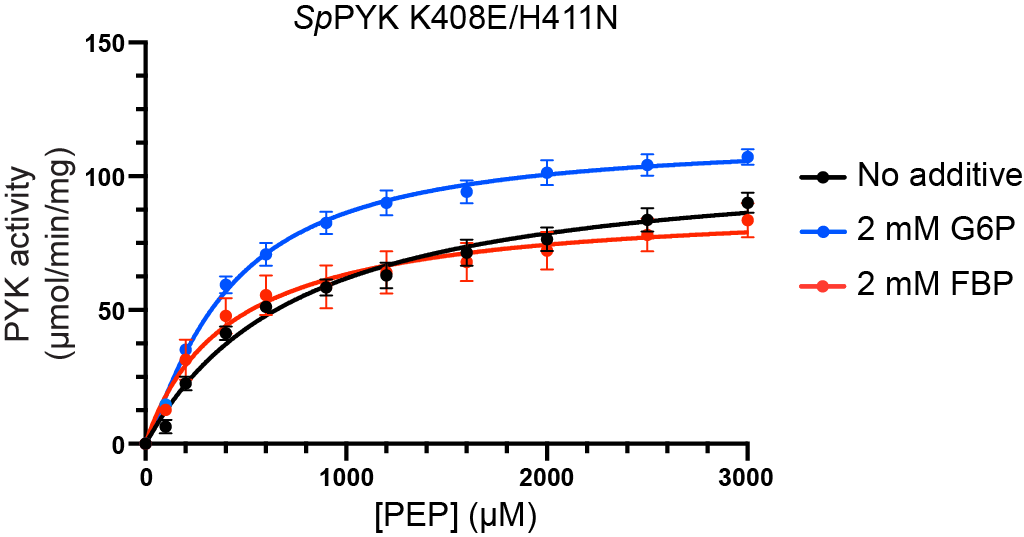
**Figure S16. *Sp*PYK(K408E/H411N) kinetics with respect to PEP.** Error bars represent mean ± SD from triplicates. See Table S2 for kinetic values.


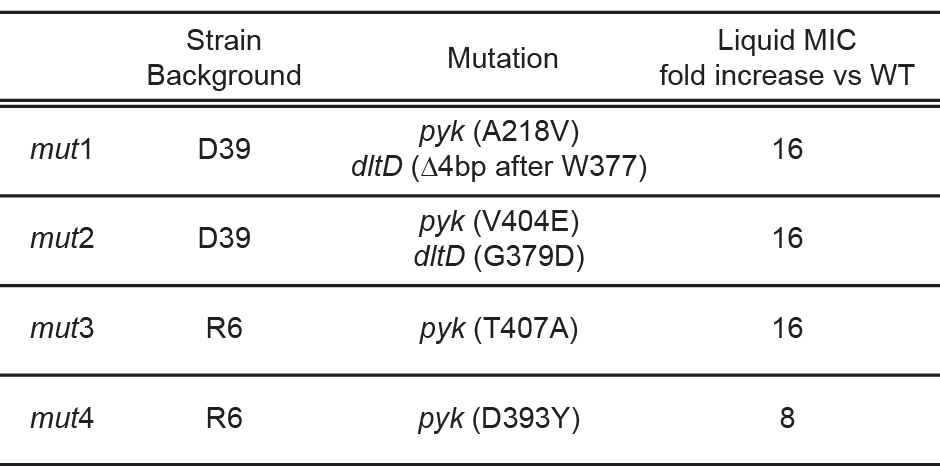


**Table S1. Fosfomycin-resistant *S. pneumoniae* strains obtained in this study.** Genetic mutations were identified by whole-genome sequencing. Fold increase in fosfomycin MIC compared to wild-type is shown for each mutant (D39 wild-type: 16 µg/mL; R6 wild-type: 32 µg/mL).


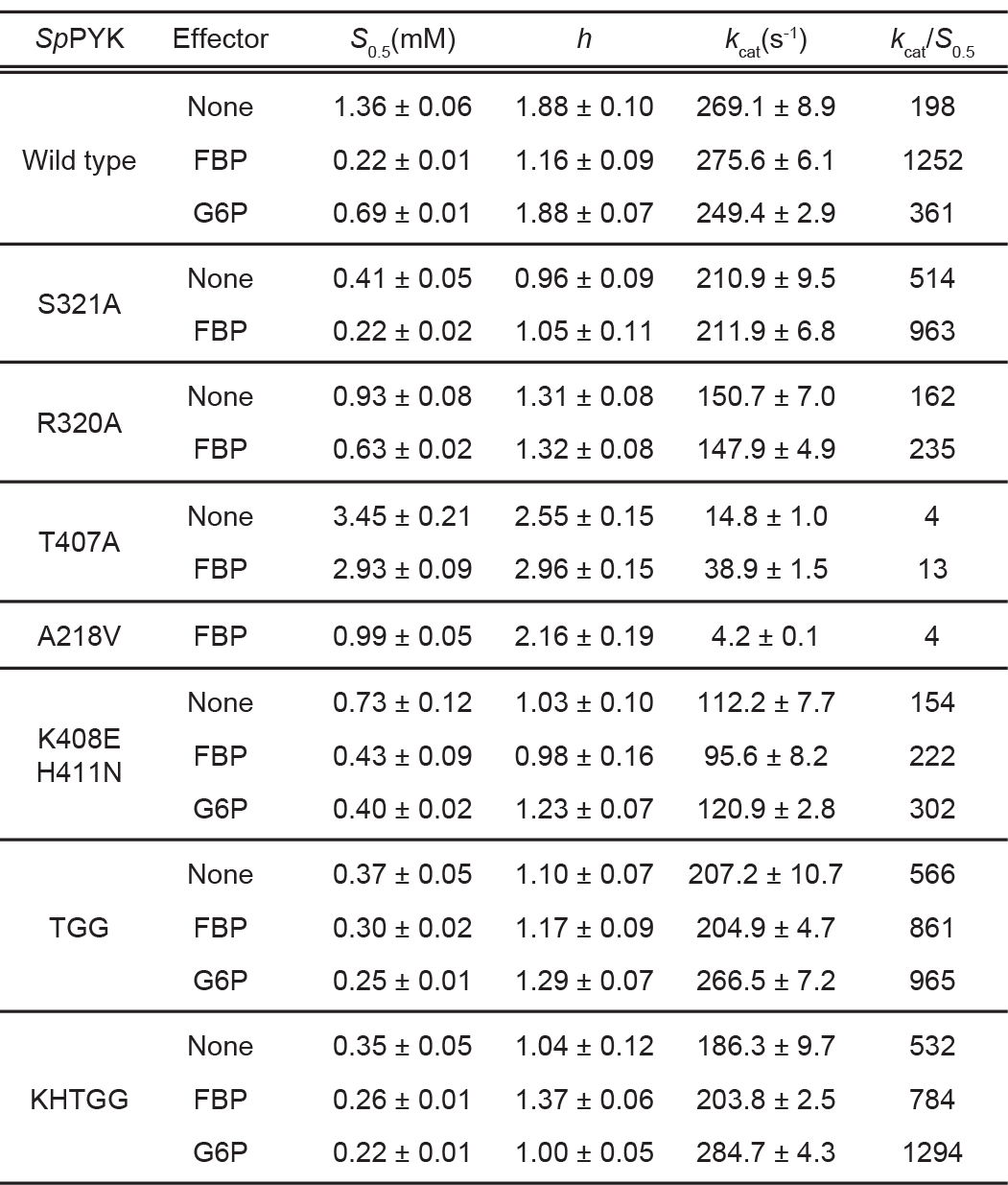


**Table S2. Kinetic data for *Sp*PYK with respect to its substrate PEP.** All data are mean ± SEM from experiments done in triplicate.

TGG: E488_R491delinsTGG

KNTGG: K408E/H411N/E488_R491delinsTGG

**Table S3. Data collection and refinement statistics for *Sp*PYK.**

|  | **Apo** | | **Oxalate** | **Oxalate/FBP** | **FBP** | **PEP** | **PEP/FBP** |
| --- | --- | --- | --- | --- | --- | --- | --- |
| PDB ID | | 8IAS | 8IAT | 8IAU | 8IAV | 8IAW | 8IAX |
| **Data collection** | |  |  |  |  |  |  |
| Light source | | BL44XU  Spring-8 | BL44XU  Spring-8 | BL44XU  Spring-8 | BL44XU  Spring-8 | BL44XU  Spring-8 | BL44XU  Spring-8 |
| Space group | | *P*1 | *C*2 | *P*1 | *C*2 | *P*2_1_2_1_2 | *C*2 |
| Wavelength (Å) | | 0.900 | 0.900 | 0.900 | 0.900 | 0.900 | 0.900 |
| Cell dimensions | |  |  |  |  |  |  |
| *a, b, c* (Å) | | 75.09 84.71 107.01 | 212.05 118.35 99.82 | 73.44 114.66 140.71 | 217.03 86.79 129.86 | 96.06 110.37 130.93 | 221.06 86.64 258.15 |
| α, β,γ (°) | | 101.22 96.82 91.42 | 90.00 108.93 90.00 | 88.95 88.13 76.52 | 90.00 103.22 90.00 | 90.00 90.00 90.00 | 90.00 100.86 90.00 |
| Resolution (Å) | | 2.00 (2.11-2.00) | 1.80 (1.90-1.80) | 2.00 (2.11-2.00) | 2.60 (2.74-2.60) | 2.90 (3.07-2.90) | 1.80 (1.90-1.80) |
| *R*_merge_ | | 8.7 (78.5) | 9.0 (86.9) | 8.0 (91.8) | 5.0 (95.4) | 17.1 (101.0) | 6.3 (102.9) |
| *I*/*σI* | | 9.78 (2.17) | 10.84 (1.91) | 8.85 (1.37) | 18.21 (1.79) | 9.68 (2.67) | 14.83 (1.81) |
| Completeness (%) | | 96.7 (96.1) | 99.7 (99.0) | 97.0 (96.4) | 99.2 (98.9) | 99.8 (99.7) | 99.6 (98.7) |
| CC (1/2) | | 99.7 (69.2) | 99.6 (87.3) | 99.7 (59.8) | 99.9 (89.0) | 99.5 (84.4) | 99.9 (80.1) |
| **Refinement** | |  |  |  |  |  |  |
| Resolution (Å) | | 49.07-2.00 | 48.30-1.80 | 49.38-2.00 | 47.69-2.60 | 48.58-2.892 | 48.37-1.80 |
| Number of reflections | |  |  |  |  |  |  |
| Observed | | 602149  (96440) | 1498593 (238202) | 1056616 (175275) | 375828  (59909) | 176383  (28461) | 3095803 (498713) |
| Unique | | 168397 (27098) | 215295 (34449) | 293893 (47226) | 72422 (11579) | 31733 (5058) | 442679 (70550) |
| *R*_work_/*R*_free_ | | 0.1781/0.2152 | 0.1987/0.2458 | 0.1994/0.2460 | 0.2319/0.2983 | 0.2408/0.2728 | 0.1825/0.2212 |
| Number of atoms | |  |  |  |  |  |  |
| Protein | | 15344 | 15369 | 29996 | 13864 | 7680 | 30720 |
| Ligand/ion | | 32 | 95 | 283 | 80 | 52 | 256 |
| Water | | 857 | 1019 | 916 | 18 | 22 | 2689 |
| B-factors | |  |  |  |  |  |  |
| Protein | | 42.21 | 48.10 | 50.09 | 107.95 | 54.07 | 42.73 |
| Ligand/ion | | 54.84 | 56.75 | 51.00 | 86.95 | 54.96 | 37.57 |
| Water | | 45.07 | 49.68 | 47.40 | 77.75 | 28.43 | 47.98 |
| RMS deviations | |  |  |  |  |  |  |
| Bond lengths (Å) | | 0.0113 | 0.0093 | 0.0117 | 0.0064 | 0.0075 | 0.0113 |
| Bond angles (°) | | 1.4651 | 1.5562 | 1.7594 | 1.4231 | 1.4766 | 1.7617 |

^*^ Highest resolution shell is shown in parentheses.

**Table S4. Bacterial strains used in this study**

| **Strain** | **Description**^*^ | **Reference** |
| --- | --- | --- |
| *E. coli* |  |  |
| XL1-Blue | Host strain for plasmid cloning | BioElegen Tech |
| ECOS Sonic | BL21(DE3) derivative strain for protein production | Nippon Gene |
| *S. pneumoniae* |  |  |
| D39 | Wild-type | S. Kawabata |
| AT1252 | D39 *pyk* (A218V)*, dltD* (∆4 bp after W377) | This study |
| AT1254 | D39 *pyk* (V404E)*, dltD* (G379D) | This study |
| R6 | Wild-type | S. Kawabata |
| AT1257 | R6 *pyk* (T407A) | This study |
| AT1258 | R6 *pyk* (D393Y) | This study |
| AT1003 | R6 *∆prsA*::(PF6-*lacI*, *gent*); Gent^R^ | This study |
| AT1023 | R6 *∆prsA*::(PF6-*lacI*, *gent*), ∆*spr1750* ::(P_lac_-*pyk*); Gent^R^, Spec^R^ | This study |
| AT1275 | R6 *∆prsA*::(PF6-*lacI*, *gent*), ∆*spr1750* ::(P_lac_-*pyk*); ∆*pyk*::(*erm*, *sacB*); Gent^R^, Spec^R^, Erm^R^ | This study |
| AT1285 | R6 *∆prsA*::(PF6-*lacI*, *gent*), ∆*spr1750* ::(P_lac_-*pyk*); ∆*pyk*; Gent^R^, Spec^R^ | This study |
| AT1405 | R6 *∆prsA*::(PF6-*lacI*, *gent*), ∆*spr1750* ::(P_lac_-*pyk*); *pyk* (S382A); Gent^R^, Spec^R^ | This study |
| AT1406 | R6 *∆prsA*::(PF6-*lacI*, *gent*), ∆*spr1750* ::(P_lac_-*pyk*); *pyk* (T384A); Gent^R^, Spec^R^ | This study |
| AT1407 | R6 *∆prsA*::(PF6-*lacI*, *gent*), ∆*spr1750* ::(P_lac_-*pyk*); *pyk* (S382A, T384A); Gent^R^, Spec^R^ | This study |
| AT1408 | R6 *∆prsA*::(PF6-*lacI*, *gent*), ∆*spr1750* ::(P_lac_-*pyk*); *pyk* (H411A); Gent^R^, Spec^R^ | This study |
| AT1409 | R6 *∆prsA*::(PF6-*lacI*, *gent*), ∆*spr1750* ::(P_lac_-*pyk*); *pyk* (R491A); Gent^R^, Spec^R^ | This study |

**Table S5. Plasmids used in this study**

| **Plasmid** | **Description**^*^ | **Reference** |
| --- | --- | --- |
| pET28b(+) | IPTG-inducible protein expression vector; Kan^R^ | Novagen |
| pATOS125 | His_6_-*pyk* expression vector; Kan^R^ | This study |
| pATOS134 | His_6_-*pyk* (T407A) expression vector; Kan^R^ | This study |
| pATOS138 | His_6_-*pyk* (A218V) expression vector; Kan^R^ | This study |
| pATOS182 | His_6_-*pyk* (S382A) expression vector; Kan^R^ | This study |
| pATOS183 | His_6_-*pyk* (S382A, T384A) expression vector; Kan^R^ | This study |
| pATOS184 | His_6_-*pyk* (T407A) expression vector; Kan^R^ | This study |
| pATOS187 | His_6_-*pyk* (H411A) expression vector; Kan^R^ | This study |
| pATOS188 | His_6_-*pyk* (R491A) expression vector; Kan^R^ | This study |
| pATOS194 | His_6_-*pyk* (S321A) expression vector; Kan^R^ | This study |
| pATOS195 | His_6_-*pyk* (R320A) expression vector; Kan^R^ | This study |
| pATOS198 | His_6_-*pyk* (K408E, H411N) expression vector; Kan^R^ | This study |
| pATOS199 | His_6_-*pyk* (E488_R491delinsTGG) expression vector; Kan^R^ | This study |
| pATOS200 | His_6_-*pyk* (K408E, H411N, E488_R491delinsTGG) expression vector; Kan^R^ | This study |
| pPEPY-PF6-lacI | *S. pneumoniae prsA::*PF6-*lacI* integration vector; Gent^R^, Kan^R^ | (11), Addgene |
| pPEPZ-P_lac_ | *S. pneumoniae spr1750* integration vector containing IPTG-inducible P_lac_ promoter; Spec^R^ | (60), Addgene |
| pATOS133 | *S. pneumoniae spr1750*::P_lac_-*pyk* integration vector; Spec^R^ | This study |

^*^Abbreviations: Erm^R^, erythromycin resistance; Gent^R^, gentamicin resistance; Kan^R^, kanamycin resistance; Spec^R^, spectinomycin resistance

**Table S6. Oligonucleotide primers used in this study**

| **Primer** | **Sequence (5’-3’)**^*^ |
| --- | --- |
| oAT201 | AGTACATATGAACAAACGTGTAAAAATCGT |
| oAT202 | ACTGGATCCTTAACGTACTGTGCGGATAC |
| oAT203 | AGCGTAACGCTAAGACAGAAGTAATGGCTTCTGC |
| oAT204 | TCTTAGCGTTACGCTCAAATGAATCTGAATC |
| oAT205 | ACTCTAAGGCAGAAGTAATGGCTTCTGCTGT |
| oAT206 | CTTCTGCCTTAGAGTTACGCTCAAATGAATCT |
| oAT207 | GACAGGTGCTACTGCACGTTTGATTTCTAAATAC |
| oAT208 | GCAGTAGCACCTGTCTTAGTAAGAGTTACAACC |
| oAT209 | AGCTGTTGCCACAAACACAATGCGTATCCG |
| oAT210 | TTTGTGGCAACAGCTTCTCCTACTGGC |
| oAT211 | GTAACGCTAAGGCAGAAGTAATGGCTTCTGCTGT |
| oAT212 | CTGCCTTAGCGTTACGCTCAAATGAATCTGAATCA |
| oAT213 | AACTCGTGCAGAAGTATCAGACGTATTCAA |
| oAT214 | ACTTCTGCACGAGTTGCACGTGGTTTTTCA |
| oAT215 | TGCAACTGCTTCAGAAGTATCAGACGTATT |
| oAT216 | TCTGAAGCAGTTGCACGTGGTTTTTCAGTC |
| oAT217 | TATTTTTCCTCCTTATTTATTTAGATCTTAATTGTG |
| oAT218 | GATCCCTCCAGTAACTCGAG |
| oAT219 | TAAGGAGGAAAAATAATGAACAAACGTGTAAAAATCGT |
| oAT220 | GTTACTGGAGGGATCTTAACGTACTGTGCGGATAC |
| oAT221 | AGAAAAAGGAGCATAAACCAATA |
| oAT222 | CTGTCTTTGATATCATCGGAGATTTTTGTGACTCCTTTATATAT |
| oAT223 | GAGAGCACAGATACGGCGATATCCAGCTTTCTAGCCTGTA |
| oAT224 | TCTCCTAGCGATATCCAATC |
| oAT225 | TCCGATGATATCAAAGACAGATTGAAA |
| oAT226 | CGCCGTATCTGTGCTCTC |
| oAT227 | GATTTTTGTGACTCCTTTATATAT |
| oAT228 | ATATATAAAGGAGTCACAAAAATCATATCCAGCTTTCTAGCCTGTA |
| oAT229 | ATATATAAAGGAGTCACAAAAATCATGAACAAACGTGTAAAAATCGT |
| oAT230 | CAGGCTAGAAAGCTGGATATGATAGGTTTTTATATTTTTCTTAACGTACTGTGCGGATAC |
| oAT231 | ATATCCAGCTTTCTAGCCTGTA |
| oAT232 | ACAACCCACACGAAGCCG |
| oAT233 | CCATTATGAAAGTTTTGCCC |
| oAT234 | ACTGAGACAGGTAATACTGCACGTTTGATTTCT |
| oAT235 | ATTACCTGTCTCAGTAAGAGTTACAACCAATTTGA |
| oAT236 | AGGAACCGGAGGAACAAACACAATGCGTATCCGCA |
| oAT237 | GTTCCTCCGGTTCCTACTGGCACACCAGCA |

^*^ Underlined sequences are restriction sites introduced in primers.
